# An *ex vivo* Approach to Study Hormonal Control of Spermatogenesis in the Teleost *Oreochromis niloticus*

**DOI:** 10.3389/fendo.2020.00443

**Published:** 2020-07-10

**Authors:** Michelle Thönnes, Marlen Vogt, Katja Steinborn, Krist N. Hausken, Berta Levavi-Sivan, Alexander Froschauer, Frank Pfennig

**Affiliations:** ^1^Faculty of Biology, School of Science, Institute of Zoology, Technische Universität Dresden, Dresden, Germany; ^2^Department of Animal Sciences, The Robert H. Smith Faculty of Agriculture, Food, and Environment, Hebrew University of Jerusalem, Rehovot, Israel

**Keywords:** spermatogenesis, testis culture, Nile tilapia (*Oreochromis niloticus*), FSH, LH, hCG, pituitary extract, Amh (anti-Müllerian hormone)

## Abstract

As the male reproductive organ, the main task of the testis is the production of fertile, haploid spermatozoa. This process, named spermatogenesis, starts with spermatogonial stem cells, which undergo a species-specific number of mitotic divisions until starting meiosis and further morphological maturation. The pituitary gonadotropins, luteinizing hormone, and follicle stimulating hormone, are indispensable for vertebrate spermatogenesis, but we are still far from fully understanding the complex regulatory networks involved in this process. Therefore, we developed an *ex vivo* testis cultivation system which allows evaluating the occurring changes in histology and gene expression. The experimental circulatory flow-through setup described in this work provides the possibility to study the function of the male tilapia gonads on a cellular and transcriptional level for at least 7 days. After 1 week of culture, tilapia testis slices kept their structure and all stages of spermatogenesis could be detected histologically. Without pituitary extract (tilPE) however, fibrotic structures appeared, whereas addition of tilPE preserved spermatogenic cysts and somatic interstitium completely. We could show that tilPE has a stimulatory effect on spermatogonia proliferation in our culture system. In the presence of tilPE or hCG, the gene expression of steroidogenesis related genes (*cyp11b2* and *stAR2*) were notably increased. Other testicular genes like *piwil1, amh*, or *dmrt1* were not expressed differentially in the presence or absence of gonadotropins or gonadotropin containing tilPE. We established a suitable system for studying tilapia spermatogenesis *ex vivo* with promise for future applications.

## Introduction

Production of functional, haploid sperm is a major prerequisite for male reproduction. Spermatogenesis starts with undifferentiated spermatogonia that proliferate by mitotic divisions before they enter the meiotic stage and differentiate into spermatocytes. After meiosis II, the spermatocytes differentiate into spermatids, which change their morphology drastically during spermiogenesis and finally become mature spermatozoa. Some fish species have a seasonal reproductive cycle while others preserve their fertility all year round. In such non-seasonal breeders, for example the Nile tilapia (*Oreochromis niloticus*), the availability and proliferation of spermatogonia has to be maintained continuously.

The hormones of the hypothalamus-pituitary-gonadal axis (HPG axis) centrally regulate reproduction. The hypothalamus produces gonadotropin releasing hormone (GnRH) which triggers the release of heterodimeric gonadotropins follicle stimulating hormone (FSH) and luteinizing hormone (LH) from the pituitary gland (1, 2). The hormonal signals from the pituitary are mediated by receptors primarily expressed on the somatic cells in the gonads, namely Leydig cells and Sertoli cells, or the germline (3). These cells then regulate further processes such as steroidogenesis or germ line cell proliferation (4, 5). While in mammals the receptors for LH (LHCGR) and FSH (FSHR) are discretely expressed in either Leydig cells or Sertoli cells, respectively, in several fish species these cells possess both LHCGR and FSHR, for example zebrafish (6), African catfish (7), Japanese eel (8), and salmon (9). Moreover, gonadotropin interactions with their corresponding receptors are very specific in mammals, suggesting that each hormone has a distinct function. This functional separation is less clear in fish due to the cross-activation of the FSHR by LH in several species (6, 10). Gonadotropin knockout models further emphasize this complication in differential signaling. Chu et al. (11) showed that male zebrafish fertility was not affected by knockouts of the *lhb* or *fshb* beta subunits or *lhcgr* or *fshr*. Fertility was only lost in double mutants lacking either both receptors or the beta subunits for both hormones (12). Zhang et al. (13) further showed that *fshr* knockout in zebrafish females lead to follicular arrest and sex-reversal into fertile males. In Nile tilapia, the specificity of the gonadotropin receptors is also debated. Aizen et al. (14) claimed the tilapia LHCGR and FSHR to be specific but a recent study showed activation of medaka and tilapia gonadotropin hormone receptors with heterologous gonadotropins (15). All of our data suggest that tilapia LHCGR and FSHR are specific for their cognate ligands (own unpublished results) and the situation in Nile tilapia could be different to that in zebrafish where FSH and LH can cross activate the respective receptors (12, 16). Taken together, the gonadotropin receptor situation in teleosts is not completely unraveled yet.

One of the cell types susceptible to gonadotropin signaling are Sertoli cells, which are part of the tubular compartment in teleost testes. They form the walls of the cysts in which synchronized development of spermatogonia takes place and they regulate spermatogenesis (2). The Anti-Müllerian hormone (AMH) is one of the hormones secreted by the Sertoli cells. In mammals, AMH causes regression of the Müllerian ducts during male sexual differentiation. An AMH ortholog exists in teleosts, playing an important role in male development and spermatogenesis, but the eponymous Müllerian ducts are not developed. In adult teleosts, Amh is thought to inhibit the proliferation of spermatogonia and their transition into meiotic spermatocytes (17–20). The first teleost *amh* gene was discovered in the Japanese eel (*Anguilla japonica*) and named eel spermatogenesis related substance (eSRS21) because it inhibited the onset of spermatogenesis. When hCG was injected, transcription of eSRS21 was inhibited and spermatogenesis ensued (21). Heterogeneous effects on *amh* expression have been reported in reaction to androgens, estrogens, gonadotropins, cortisol, and temperature for different teleost species (22). Most studies on teleost Amh regulation come from zebrafish, where FSH was found to down-regulate *amh* expression in adult testis (19, 23, 24). While models for these regulatory networks have been proposed, the target genes of Amh are still mostly unknown (25, 26).

Organ culture systems have the advantage that they present a middle way between primary cell culture and *in vivo* experiments. The intercellular connections and tissue specific environment stay intact and can be manipulated without affecting the live animal prior to sampling. For zebrafish and Japanese eel, there are well-established testis culture protocols available (27, 28) where the tissue is not submerged in the medium directly but connected to it by a nitrocellulose membrane on top of an agarose block. Unfortunately, these procedures have limitations in other species like the Nile tilapia, where a similar approach has only been reported for juvenile gonads from fry (29). Although short-term stationary culture in well-plates is possible, long-term cultivating systems are needed to study the complete process of spermatogenesis in adult tilapia. The duration of the spermatogenic process in tilapia is dependent on temperature. At 25°C it takes 10–11 days for spermatocytes to develop into spermatozoa, whereas at 30°C this time span shortens to about 7 days (30, 31). Reliable data about the duration of fish spermatogonia development during the early phase of spermatogenesis are not available. Because oogonia and spermatogonia are very similar at their early development (32), we refer to a study about the dynamics of medaka oogonia proliferation (33). In medaka ovaries it was shown that fast cycling early oogonia need 37 h to complete one cell cycle (33). From catfish it is known that type B spermatogonia (SgB) proliferate up to 5 times faster than type A spermatogonia (SgA) (17). When taking those data from medaka and catfish as a rough reference, tilapia spermatogonia could need about 5–7 days to complete their 7 mitotic division before entering meiosis (4).

In this study, we established a long term, closed, flow-through culture system with circulating medium in order to study tilapia spermatogenesis until the meiotic stage. This system provides sufficient oxygen and nutrients for the testis slices to enable a functional culture for 1 week. This newly established culture system facilitated the aim of this study, which was to investigate the performance of tilapia testis explants with and without gonadotropic stimuli. To this end, we used native hormones from tilapia pituitary extract (tilPE) and the potent LH analog, human chorionic hormone (hCG). Methods to evaluate the treatment effects included studies of gene expression of testicular marker genes (like the putative FSH target *amh*), histology, and proliferation assays.

## Materials and Methods

### Fish Maintenance and Sampling

The animals used in this study were bred and reared in the institute's fish facility. All specimens originated from a brood stock of *Oreochromis niloticus* L. brought to Dresden in 2004 from the Leibniz-Institut für Gewässerökologie und Binnenfischerei Berlin, Germany. Purebred *O. niloticus* came in 1994 to Berlin from the “Research Institute of Fish culture and Hydrobiology” in Vodnany, Czech Republic (personal communication Bernhard Rennert, Leibniz-Institut für Gewässerökologie und Binnenfischerei Berlin, Germany). Fish in our facility were housed in 550 or 820 L tanks in a recirculating system at 26°C and a 14 h light: 10 h dark photoperiod. Five percent of the water was exchanged daily and electric conductivity was around 600 μS/cm. Commercial food (Pro Aqua Brut 1.0 MP, Skretting, Norway) was fed twice a day *ad libitum*, until the fish reached 6 months of age. Then, one family group of 20–30 siblings of the same brood was fed daily with F3-P Optiline (6 mm, Skretting, Norway), supplemented with vitamins (multivit, hw-Wiegand, Germany) twice a week. Once a month trace elements (tracevit, hw-Wiegand, Germany) were added to the water directly.

For the experiments described in this study, we used adult males between 13 and 55 months of age. These animals had normally developed testes with GSIs (gonadosomatic index) ranging from 0.14 to 0.32, which are typical values for fish of this age and weight, as has been established by Pfennig et al. (34). The excretion of milt and the histological data were taken as proxies for intact spermatogenesis in these testes. The GSI of all fish in this study are listed in Table S1.

The 7-day culture for the gene expression experiment was repeated 4 times in total. In three experiments gonad slices from two fish were used and in one experiment tissue from one male was used. From two of those runs, proliferation assays with EdU were performed. Very socially low ranking animals, or such with underdeveloped testes were excluded from the experiments.

Experimental animals were anesthetized in 0.2 g/L benzocaine (Sigma, USA). After weighing and measuring, the fish were killed by severing the spinal cord with a pair of scissors. The testes were weighed for the GSI and rinsed in sterile PBS to remove seminal fluid, then washed in Hank's solution (Biochrom, Germany) before the transfer into the culture chambers. Testes slices to be used for gene expression analysis were snap frozen in liquid nitrogen and stored at −80°C until RNA extraction.

Animal culture and euthanasia were performed according to the German national regulations and animal welfare. Facilities and procedures are approved by administrative regulations (Regierungspräsidium Dresden 24D-9165.40/8-2006-1).

### Preparation of Pituitary Extract (tilPE)

To prepare the extract, pituitary glands from *O. niloticus* were first incubated in 1.5 ml of absolute ethanol for 24 h at room temperature. The ethanol was changed and the pituitaries were incubated for another hour. After removing the ethanol, the glands were dried shortly on an absorbent tissue. Alternatively, the pituitaries were dried in acetone. In this case, the fluid was changed after 24 h and 30 h. In a next step, the samples were air-dried, weighed, and transferred into sterile PBS. Then, the glands were macerated mechanically using a pestle. After 1 h of incubation on ice, the extract was separated from the pellet by centrifugation (5 min at 4,000 × g). For all experiments a concentration of 0.1 mg/ml (dry weight) tilPE was used. The pituitary glands used for extract preparation were mainly those of spare female animals from our stock. The content of FSH and LH was measured by ELISA (35). The gonadotropin content of the pituitaries was 4 μg FSH/ pituitary and 7.5 μg LH/ pituitary. Therefore, the final hormone concentrations in the medium were 430 ng/ml FSH and 780 ng/ml LH. These are high values when compared to plasma levels measured in fish from our facility for another experiment where dominant males reached 37 ng/ml FSH and 20 ng/ml LH. Unlike in a living animal, blood flow is not existent in the culture system. Exchange of substances occurs via diffusion. To make sure, the organ still receives sufficient levels of tilPE, we used comparatively high concentrations of it in the medium.

### *Ex vivo* Culture System

**Figure 1:**
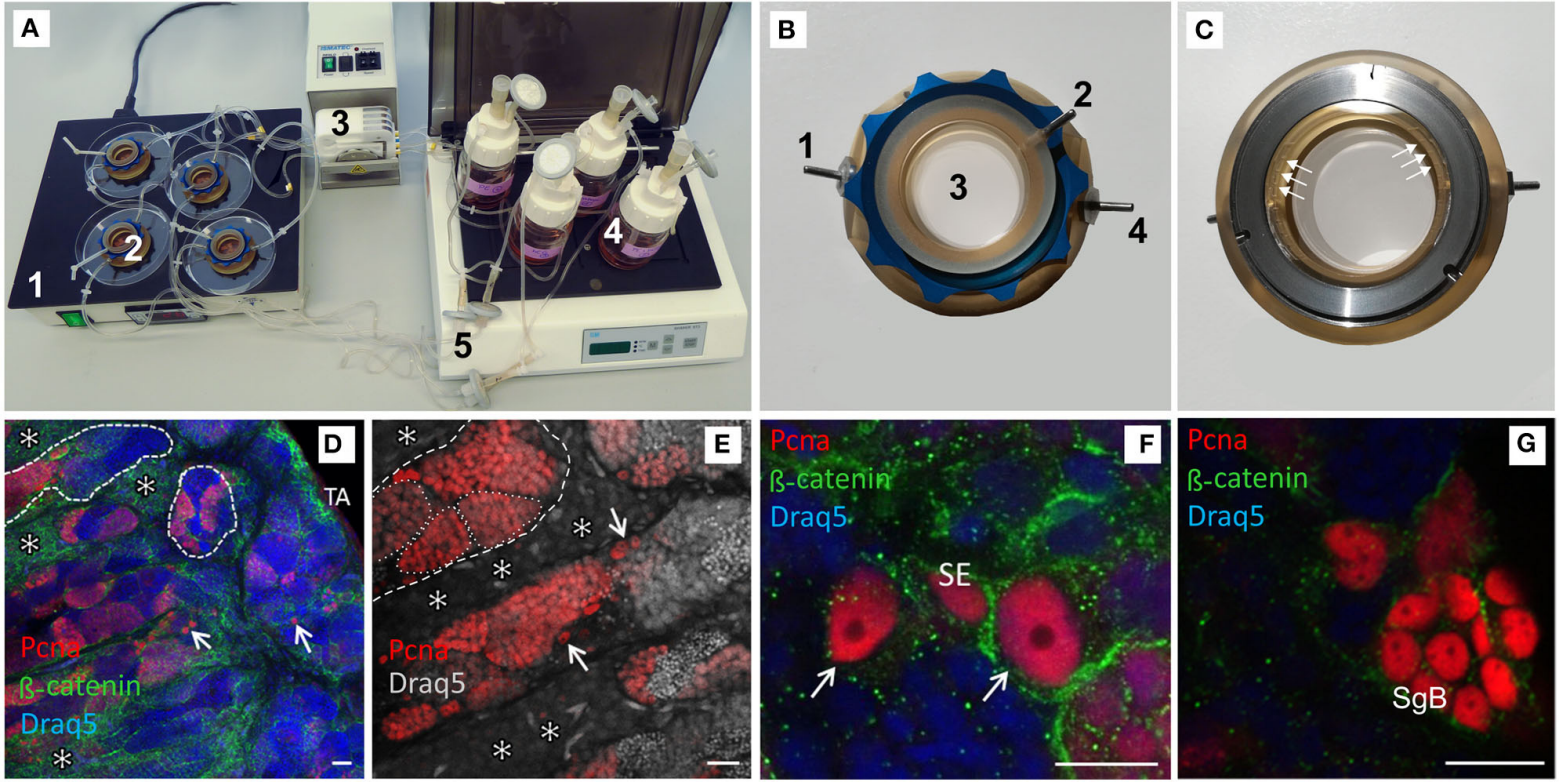
Confocal images of vibratome sections of testis explants cultured for 7 days in a flow-through chamber and activated by pituitary extracts. **(A)** Picture of complete flow-through culture system; **1**—temperature unit, **2**—chambers, **3**—peristaltic pump, **4**—media bottles, **5**—connecting tubes with sterile filter in the backflow. **(B)** Chamber top view, **1**—inflow, **2**—vent, **3**—medium filled part of the chamber, **4**—outflow. **(C)** Chamber bottom view, white arrows mark the access channels to the in- and outflow exemplarily (3 out of 10). **(D)** Intact testis morphology with spermatogenic tubules (dashed lines), proliferating germ line cells in cysts (red label by Pcna antibody), single Pcna positive type A spermatogonia (arrows), well developed interstitium with Leydig cell groups (*), tunica albuginea (TA); DNA (Draq5 in false color)—blue, for visualization of cellular size and shape β-catenin antibody (green) was used—preferably type A spermatogonia and Leydig cells were stained. **(E)** Many Pcna positive germ line cells in cysts (exemplary labeled by a dotted line) and single type A spermatogonia (arrows). **(F)** Pcna-positive, undifferentiated type A spermatogonia (identification by nuclear morphology, arrow) adjacent to a Pcna positive Sertoli cell (SE). **(G)** Cyst with Pcna expressing type B spermatogonia (SgB). Scale bars: 20 μm in **(D,E)**, 10 μm in **(F,G)**.

Each testis was cut into transversal sections of ~2 mm thickness for culture. Only sections from the middle part of the testis were chosen to account for varying cell composition along the testis strand. In the first of three replicated experiments 3 pieces from one male fish were used in every condition. In the other two replicates, 3 sections from each of the two males were cultured under the same condition in special flow-through chambers. The chambers were based on those described by Gutzeit and Kaltenbach (36). These chambers were developed further with and manufactured by Thalheim Spezialoptik GmbH (Germany, thalheim@tso-optik.de). They were connected with the aerated medium supplies and a peristaltic pump (Reglo Analog ISMB827B from Ismatec, Germany). The flow rate was set to 3 ml/min and the medium (supplemented with hCG, tilPE, hCG + tilPE, or DMSO) volume was 30 ml. The culture medium consisted of L15 Leibowitz medium (Biochrom, Germany) supplemented with 10% FBS (fetal bovine serum, FBS Superior, Biochrom, Germany), 2.5 μg/ml Amphotericin (Biochrom, Germany), and 0.1 mg/ml Gentamycin (Biochrom, Germany). To provide optimal culture conditions the chambers were placed on a heating table set to 26°C (corresponding to the water temperature in which the fish were housed). The experimental setup is shown in Figures 1A–C.

### RNA Isolation and cDNA Synthesis

Total RNA was extracted from the organ pieces after cultivation using peqGOLD TriFast (PEQLAB-Lifescience, Germany) according the manufacturer's protocol. The organ pieces were macerated by hand with 1.5 cm micro-pestles (USA Scientific, USA). For RNA quantification a NanoDrop 1000 Spectrometer (Thermo Fischer Scientific, USA) was used, and RNA integrity was assessed on a 1.5% agarose gel. RNA samples were treated with DNAseI (recombinant, DNase free from Roche, Switzerland) to digest any contaminating gDNA.

Total RNA (2 μg) was reverse transcribed into cDNA using the High-Capacity Reverse Transcription Kit (Thermo Fisher Scientific, USA) with random hexamers according to the manufacturer's protocol. Successful cDNA synthesis was validated with β*-actin* PCR (Table S2). The β*-actin* primers binding sites are separated by an intron, allowing discrimination between genomic DNA and cDNA products.

### RT-qPCR

Quantitative real-time PCR was performed using a QuantStudio 5 Real-Time PCR System (Thermo Fisher Scientific, USA). The reactions were carried out in a total volume of 10 μl, containing 5 μl PowerUp SYBR Green Mastermix (Thermo Fisher Scientific, USA), final primer concentrations of 250 nM, and 22.5 ng cDNA per well. The reaction was run in triplicate for each sample. The thermocycler was used with the following parameters.

50°C for 2 min, 95°C for 10 min, followed by 40 cycles of 95°C for 15 s and 60°C for 1 min, and a final dissociation step at 95°C for 1 min. A no template control was always included for each primer set. Standard curves with serially diluted PCR product were used to check primer efficiencies, which were all between 91 and 109%. The gene expression was calculated after the method published by Pfaffl (37). Gene expression was normalized to the reference gene *18S*. All primers used for RT-qPCR are listed in Table S2.

### Histology

Testis sections of ~2 mm were fixed for 30 min in 2% PFA/ HEPES (100 mM) at RT, overnight in 4% PFA/HEPES at 4°C, and were finally stored in 1% PFA/HEPES at 4°C. To prepare the organs for vibratome sectioning, they were washed in PBS and embedded in 3% agarose blocks. After the agarose had hardened, the blocks were cut with a Hyrax V50 vibratome (Zeiss, Germany) with the following settings: slice thickness 200–250 μm, amplitude 0.3, trimming 40, frequency 60 Hz, blade angle 3°. The slices were stored in 1% PFA/HEPES for further processing.

The primary antibodies used (Table 1) are not derived from Nile tilapia proteins directly. The specificity of the anti-PCNA- and anti-Vasa-antibodies was already shown in our previous publications for testis and ovary of Nile tilapia (34, 40). The used PCNA antibody (PC10, catalog number MAB424, LOT 1766464) is a commercial mouse monoclonal antibody against the rat-PCNA protein and is well-established in a broad range of species in the animal kingdom from vertebrates to invertebrates. The polyclonal rabbit anti-Vasa-antibody (K12-3) was generated by immunization with the C-terminal part of the zebrafish Vasa protein [148aa amino acids from Position 544 to stop (38)]. The homology to the Nile tilapia Vasa at this part is very high and consensus is around 80%. Western blots showed the exact size for the two Nile tilapia isoforms [(41); data not shown]. Its cell type specificity for Nile tilapia germ line cells in the testis and for the ovary was previously shown (34, 40). The *Xenopus* epitope P14L [PGDSNQLAWFDTD (39)], which was used to generate the rabbit polyclonal anti-β-catenin-antibody, matches 100% with the corresponding Nile tilapia β-catenin1 and fits β-catenin2 (42). The β-catenin antibody is well-characterized in *Xenopus* (39, 43) and works also in zebrafish (44).

**Table 1:** Dilutions of primary and secondary antibodies used in this study.

	**Host**	**Function**	**Dilution**	**Source**
**PRIMARY ANTIBODIES**				
Anti PCNA PC10 (MAB 424)	Mouse	S-phase marker	1:50/1:100	MerkMillipore, Germany
	(monoclonal)			
ZF Vasa K12	Rabbit	Detection of spermatogonia	1:100	Knaut et al. (38),
	(polyclonal)			from Schwarz, Tübingen, Germany
Anti-β-catenin (P14L)	Rabbit	Visualization of general testis structure	1:10	Schneider et al. (39),
	(polyclonal)			from Kurth, Dresden, Germany
**SECONDARY ANTIBODIES**				
Alexa Fluor 488 #4408	Goat	Anti-mouse	1:200	Cell Signaling Technology, Germany
660 C SAB4600195	Goat	Anti-rabbit	1:500	Sigma Aldrich, Germany

For Pcna staining, demasking of the samples by heating them in citric acid buffer was performed. Thus, the cross links formed between proteins by PFA fixation get broken and antigen binding sites are made accessible once again. In the case of the Pcna antibody demasking is necessary to get a signal. The samples were permeabilized (0.5% TritonX blocking buffer) for 20 min at room temperature. Blocking was performed overnight at 4°C with 20% native goat serum in PBS/0.1% Triton X. This was done to reduce unspecific binding of the antibodies.

Primary antibodies were diluted with blocking buffer to the desired concentrations (Table 1) and incubation was carried out for 48 h at 4°C. Next, the samples were washed with PBS 3 × 5 min, 1 × 15 min, 1 × 30 min, 2 × 60 min, and once for 120 min. The incubation with the secondary antibodies (diluted in blocking buffer, Table 1) lasted 24 h, after which the samples were again washed. Finally, the testis sections were post-fixed for 1 h in 4% PFA in HEPES. Before storage, the samples were shortly washed and moved to 1% PFA in HEPES.

DAPI (ROTH, Germany, 0.05 μg/ml f.c.) or Draq5 (biostatus, UK, 5 μM f.c.) was used for nuclear staining and added to the samples 1–2 h before microscopy.

### Proliferation Assay

To detect proliferating spermatogonia the Click-iT™ EdU Alexa Fluor™ 594 Imaging Kit (Thermo Fisher Scientific, USA) was used. The thymidine-analogon was added 24 h before the end of the experiment and all steps were carried out according to the manufacturer's protocol. PFA-fixed pieces from the cultivation experiment were cut with a vibratome into slices with a thickness between 200 and 250 μm. These were then immunologically stained for the germline marker Vasa and the proliferation associated DNA ring clamp protein Pcna. After Click-iT™ detection of the EdU molecules incorporated into proliferating cells during the last 24 h of cultivation, the samples were evaluated using a confocal laser scanning microscope (LSM). Three to five z-stacks (1.5 or 2.5 μm between optical slices) of 3–4 testis slices per condition were evaluated for this study.

### Microscopy

The testis sections were analyzed with a confocal laser scanning microscope (AxioObserver LSM880 or LSM700, Zeiss, Germany) and the Zen 2.3 software (Zeiss, Germany). Images were taken at an interval of 1.5–2.5 μm covering 20 and 50 μm in total. From the selected z-stacks, spermatogonia were evaluated concerning their total number and proliferation signals (Pcna or EdU fluorescence).

### Categorizing and Counting Spermatogonia

The evaluated areas of the testis sections were located close to the *tunica albuginea*, where most SgA and early SgB were found. Schulz et al. (4) reported seven rounds of mitotic division for Nile tilapia, and that the actual number of cells deviated <10% from that predicted for the stages (2^n^). Their model was consulted to evaluate the data in this study, discriminating only between SgA and SgB without regard to their differentiation status. A cell with a nuclear diameter ≥8 μm was classified as SgA and SgB were defined by a nuclear diameter between 8 and 5 μm. Besides nuclear morphology, Vasa staining facilitated the identification of spermatogonia. Vasa is an early germ cell marker, expressed predominantly in spermatogonia and weakly in primary spermatocytes, but not at later stages (41, 45). To prevent overestimation of cell numbers by counting each spermatogonium more than once we analyzed whole z-stacks. This way it is possible to mark a nucleus as belonging to one and the same cell. Using z-stacks with narrow spacing between the layers also facilitates measuring the nuclear diameter at its widest.

### Statistics

*X*^2^-test was performed in R (Version 3.5.1) to test for altered proliferation of treated and untreated spermatogonia.

Wilcox rank-sum test was applied to test for significant differences in gene expression between treatments and control. The test was performed in R (Version 3.5.1) with the default settings (two-sided approach). *w* and *p*-values for all tests performed are provided in Table S3.

## Results and Discussion

### 7-day Culture of Testis Explants Requires Addition of tilPE for Maintenance of Intact Testis Structure

In preliminary experiments with static cultures, we observed degeneration of testis structure (Figure S1 and data not shown). In order to cultivate explants for at least 1 week without loss of structure, next we used flow-through cultures in specially developed flow-through chambers with medium re-circulation (Figures 1A–C). After 7 days of culture under such constant circulation of the medium with and without gonadotropin containing tilPE or other hormone supplementation, we analyzed structural differences between the different conditions. In our histological analyses, antibodies against Pcna were used for detection of proliferative testis cells, and antibodies against β-catenin, to visualize cellular structure of the fish testis (Figures 1D–G). Although Pcna is expressed during late G1 phase and early G2 phase, its presence is strongest during the S-phase of the cell cycle and marks clearly proliferative cells (46). β-catenin has different functions and localizations in a cell. It binds cadherins in the cell membranes as a cytosolic binding protein and therefore often shows a membrane associated signal with different intensities. Further, β-catenin is a central part of the canonical Wnt-signaling pathway and can be found in the cytoplasm or in the nucleus, depending on the activity of the complex Wnt-pathway (47). In our work the goal was the illustration of shape and size of cells in the fish testis, not the evaluation of β-catenin as a mediator of Wnt signaling. In normally developed testis, the strongest membrane and intracellular signals were observed for SgA. The membrane-localized signal intensity can vary and decreased with ongoing differentiation in further advanced SgB. Reduced signal intensity appears as a granular pattern and that is the only signal still detectable in spermatocytes. Post-meiotic germ cells have no β-catenin signal. Further, a typical mesh-like pattern of membrane-associated β-catenin in groups of Leydig cells can be seen. Recently, β-catenin transcripts were detected also in Nile tilapia testis (42), and for zebrafish it has been shown that Igf3-triggered β-catenin signaling is part of the complex regulative network controlling spermatogonial differentiation (26). Both studies indicate that β-catenin can play a role in fish testis and the protein should be expressed in Nile tilapia testis.

**Figure 2:**
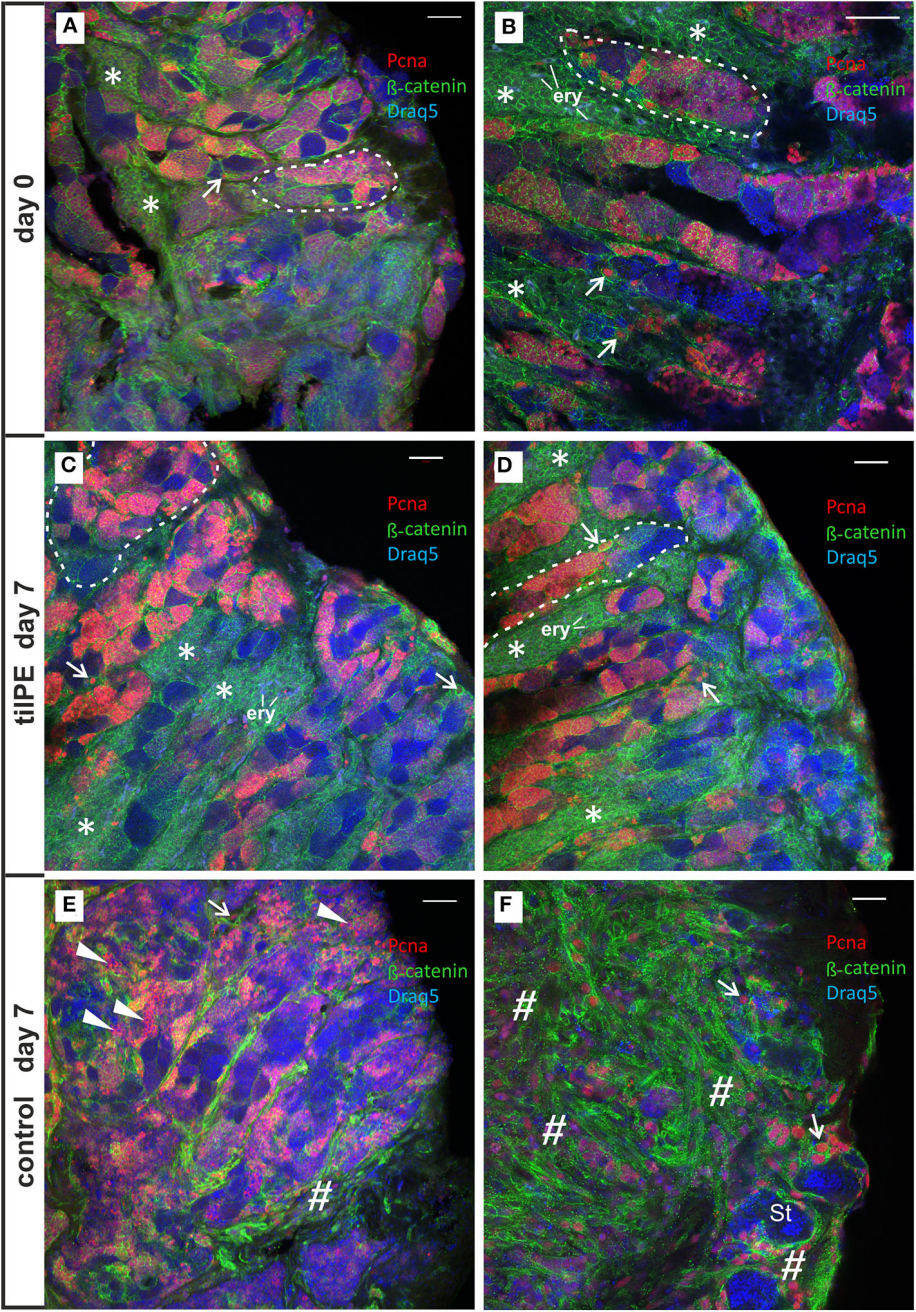
Confocal images of Pcna (red) and β-catenin (green) immunolabeled vibratome sections of 7 days cultivated testis explants. **(A,B)** Testis sections from two different explants from the beginning of the flow-through culture (day 0). Large groups of Leydig cells are indicated by an asterisk (*). Nucleated erythrocytes (exemplary labeled with “ery”) inside of these groups appear in a light gray-blue coloration (shown in higher magnification in Figure S3). Spermatogenic tubules contain cysts with cells of all stages of spermatogenesis. Abundant Pcna labeling of “single” SgA (exemplarily shown by a white arrow) and cyst consisting of SgB and spermatocytes occurred. Postmeiotic germ line cells show no Pcna and appear in blue by Draq5 stained nuclei inside of the tubules. Some spermatogenic tubules are labeled by a white dashed line exemplarily. **(C,D)** Testis sections from two different explants show completely preserved testis tissue after 7 days of flow-through culture with tilPE supplementation. Many Pcna labeled cyst cells, Pcna labeled type A spermatogonia and large groups of Leydig cells (*) are present. The interstitium appears unchanged in comparison to the start of the culture at day 0. **(E,F)** Testis sections from two different explants after 7 days of flow-through culture without addition of tilPE (control). Normally developed spermatogenic tubules are present. Cysts with all stages of spermatogenesis and Pcna labeling occur **(E)**. In a lot of germ cell cysts an unusual cytoplasmic Pcna localization can be seen (exemplarily labeled by flat triangles). The structure of the interstitium has changed, large Leydig cells groups are not obvious and fibrotic structures appear (exemplarily labeled by a diamond #). An example of severe fibrosis is shown in **(F)**. Here, a lot of interstitial cells are Pcna labeled and β-catenin staining differs from the mesh-like pattern of caused by the presence of Leydig cells. Remaining germ line cysts of the tubules display spermatids (St) or very early germ line cells (SgA—white arrow) only. The proportion of type B spermatogonia and spermatocytes seems to be reduced in **(E)** and these cell types have disappeared in **(F)**. A morphological comparison in higher magnification is shown in Figure S2 and further details in Figures S4, S5. In all pictures DNA is stained by Draq5 (in false color as blue). Scale bars: 50 μm.

**Figure 3:**
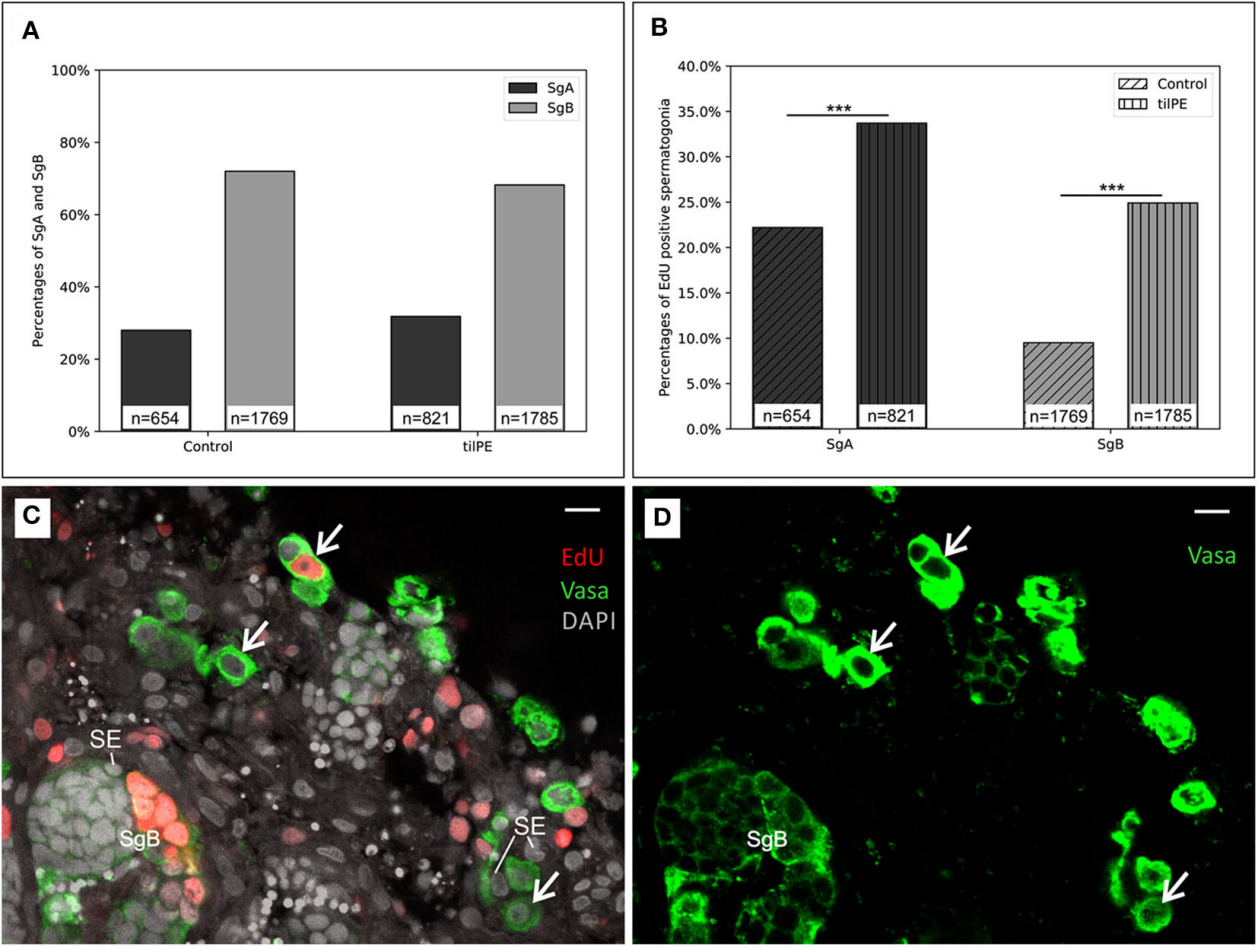
Evidence for spermatogonia proliferation. **(A)** The relative number of SgA and SgB after 7 days of organ culture. The ratio between SgA and SgB does not significantly change between the treatments. **(B)** Ratio of EdU positive spermatogonia. Compared to the control, more SgB had incorporated EdU after 7 days. EdU was added for the last 24 h of the culture. *X*^2^ test was applied: ***p < 0.001. The data displayed in **(A,B)** came from two independent experiments. Testis sections from 3 male fish were analyzed in total. **(C)** Representative testis section for spermatogonia counting (example from a 7-day culture without tilPE supplementation). Nuclei (gray) are stained with DAPI. Spermatogonia type A (white arrows) and B (SgB) were classified after nuclear diameter and Vasa expression (green). EdU incorporation (red) was used to detect proliferating cells. Sertoli cells were exemplary labeled with SE. **(D)** Green channel only of picture **(C)** to illustrate the Vasa positive cells and different intensities of the Vasa signals. Scale bar: 10 μm.

In flow-through testis cultures without additional hormone supplementation different degrees of structural alteration in testis morphology occurred after 7 days. Figures 2E,F shows such alterations side by side with the observed changes in static cultures (Figure S1). Our histological evaluation revealed spermatogenic tubules with all stages of germ line cells (Figure 2E), but in comparison to control sections from testes which were fixed directly after removal from the animal (Figures 2A,B), the number of single and cystic Pcna positive cells was reduced. In addition, the normally existing large Leydig cell islets seemed to be reduced and wide parts of the interstitium showed fibrosis, recognizable by many evenly distributed Pcna positive cells and a changed β-catenin pattern around the tubules (Figures 2E,F). Leydig cells were identified by their round nuclei, which have a diameter of almost exactly 5 μm and by their typical occurrence in groups of uniform cells outside of the spermatogenic tubules and in neighborhood with blood cells. In the remaining spermatogenic tubules or remnants of these tubules adjacent to the fibrotic areas, type B spermatogonia (SgB) were rarely seen and post-meiotic germ cells were prevalent in the cysts (Figure 2F and Figures S2, S4). SgB tend to have a higher proliferation rate than SgA (17), allowing speculation that after 7 days in culture, many of them had already become spermatocytes in our experiment. Intact areas could also be observed (Figures 2E, 3C and Figure S2).

In contrast to such observed structural loss or changes in testis morphology in cultures without hormone supplementation, the addition of Nile tilapia pituitary extracts (tilPE) to the circulating medium completely maintained testis structural integrity (Figures 2C,D). Even after 7 days of cultivation, cysts containing SgB and spermatocytes of different stages could be identified alongside single, early stage spermatogonia type A. These samples did not look different from the control slices taken at the start of the experiment (Figures 2A,B) and large groups of Leydig cells were also maintained (Figures 2C,D). Many of the germ cell cysts contained S-phase marker Pcna positive cells, indicating ongoing spermatogenesis in the testis. In contrast, the long-term culture of tilapia testis sections caused structural changes to the tissue if tilPE was not added. These findings are in accordance with the fact that missing stimulus from the pituitary gland needs to be compensated to study the process of spermatogenesis under culture conditions. Our experiment shows that tilPE is indispensable for structural maintenance of the testis under culture conditions.

When gonadotropin containing tilPE was present in the culture medium from the beginning, numerous Pcna positive cysts could be detected (Figures 1D–G, 2C,D and Figure S5). This result underlines the importance of tilPE in maintaining proliferation of spermatogonia. We could also speculate that tilPE is necessary for SgA to progress to SgB in tilapia. This observation has been made in the newt as well and is discussed in more detail below. As reviewed by Schulz et al. (2), current findings support the model that the gonadotropin FSH is involved in the transition of slow-cycling SgA, which are committed to self-renewal, toward fast-cycling SgA, which will differentiate into SgB. There are reports from seasonal and non-seasonal fish that lead to the proposition of this role for FSH. In zebrafish, germ cell proliferation was successfully increased by recombinant FSH under tissue culture conditions (48). Also using testis culture, the proliferation of SgA and SgB could be maintained up to 10 days in medaka by adding human FSH (49). In amphibians like the newt *Cynops pyrrhogaster* FSH has also been studied in regard of its role in spermatogonia proliferation. As reviewed by Uribe and Mejía-Roa (50) the mode of spermatogenesis in salamanders can best be compared to that of restricted spermatogenesis in fish. “Primary spermatogonia” seem to be analogous to SgA while SgB are referred to as “secondary spermatogonia.” In newt *ex vivo* testis culture without FSH the spermatogonia proliferated more slowly than in the presence of the hormone (51). Furthermore, secondary spermatogonia died just after their last mitotic division and before differentiating to become spermatocytes if FSH was not available. Considering the apoptotic secondary spermatogonia of newt in the absence of FSH, the study of cell death in fish SgB also appears to be an interesting focus for future research.

It is noteworthy, however, that SgA were preserved better than SgB when cultured without tilPE. As depicted in Figure 2E, many SgA showed nuclear Pcna, indicating cells in S-phase also at the end of the 7-day cultivation time of the experiment. One explanation for this observation could be that spermatogonia proliferation can be upheld independent of gonadotropins by factors further downstream the HPG axis, like 11-ketotestosterone in Japanese eel (52). Endogenous androgen production could also occur because the cultured tissue in this study contained Leydig cells. Additionally, previous work (data not shown) showed that FBS is necessary to maintain normal structure during 7-day testis cultures. The FBS used in our study contained a final concentration of 0.35 ng/ml testosterone according to the certificate of analysis. Testosterone is also an androgen in fish. The 10% proportion of FBS in the medium reduces the final concentration of testosterone to 0.035 ng/ml. Using a back calculation from zebrafish testis organ cultures (19, 28, 53), where a basal 11-KT concentration of around 1 ng per mg testis tissue was determined in the medium, then the media concentrations lays in a range of 1–2 ng 11-KT per ml. That means the FBS derived testosterone content of the medium used in our cultures was low but constituted round about 1/50 of average basal 11-KT level in zebrafish organ cultures, what seems to be sufficient for the observed preservation of SgA in the testis cultures. Apart from testosterone, the identity and concentration of growth factors in each lot of FBS is largely unknown, but there is evidence for the presence of Igf1 (insulin like growth factor1) in this product for example (54, 55). Igf1 has long been known to influence spermatogenesis in trout, *in vivo* and *in vitro* (56, 57). Treatment of tilapia testes primary cell cultures with Igf1 also stimulated proliferation of spermatogonia (58). This study used a culture medium containing 10% FBS, very similar to the one described in the present work. Nevertheless, in the Igf1 treatment caused significantly more proliferation over 16 days of the experiment than the control. Therefore, we can assume that the Igf1 contents of FBS is low enough for the tissue to stay susceptible for further stimulation. This probably holds true for other FBS components as well. As mentioned above, some stimulatory effect of FBS is desired to maintain the testis tissue during cultivation.

Another difference between tilPE and control culture regarding SgB had to do with Pcna localization. The SgB cysts in the sample without tilPE supplementation sometimes showed an unusual cytoplasmatic Pcna signal (Figure 2E and Figure S6). During S-phase, Pcna has a nuclear localization in its function as DNA ring clamp (59). The same unexpected cytoplasmic Pcna signal was observed in our static culture experiments without gonadotropic supplementation (Figures S1C–F). Possibly, cytoplasmic Pcna localization in fish testis could be connected to a role of Pcna in apoptosis as described for neutrophil granulocytes where such patterns have importance for survival of human neutrophils and HL-60 cells (60–62). This is in concordance with our observation described above, that SgB and spermatocytes made up a reduced proportion of the cells when tilPE supplementation was missing.

Finally, our experiments to establish suitable culture conditions showed that media re-circulation alone was not sufficient to preserve intact structure of testis explants for 7 days. The addition of tilPE completely maintained the active testis structure and is therefore one option to enable an experimental design spanning at least 1 week.

Based on these results, we designed follow up experiments to investigate spermatogonial proliferation and testicular gene expression in a quantitative way.

### Pituitary Extract Enhances Spermatogonia Proliferation

After 7 days the ratio of SgA to SgB was not significantly different in the untreated control or tilPE treated cultures (Figure 3A). Around 70% of the observed spermatogonia were categorized as type B and 30% were categorized as type A. These finding seems to contradict the results presented in 3.1 at first glance. However, equal proportions of SgA and SgB in treatment and control can be explained by the fact that for analysis only structurally intact areas of the testes were selected. As stated in the methods section, we applied the cell state classification by Schulz et al. (4). According to this classification, a cell with a nuclear diameter >8 μm (a criterion for SgA) should always be a single cell. However, in this study we also encountered spermatogonia with such a nuclear diameter occurring in pairs, which we labeled as SgA. The existence of SgA pairs is in concordance with other descriptions of tilapia germline cells which report clusters of SgA consisting of up to 8 cells (32). These authors classify SgA in further subgroups. The earliest SgA are called undifferentiated SgA because of their self-renewing potential. Differentiated SgA on the other hand are believed to have taken the path of further spermatogenesis (2, 32). These authors apply ultrastructural criteria like the amount of heterochromatin, the shape of the nuclear envelope or the density of mitochondria to distinguish between differentiated and undifferentiated SgA. In our study however, SgA were not subdivided into these two groups.

After comparing cell type ratios of all spermatogonia (SgA and SgB), we analyzed the numbers of such cells that were positive for EdU incorporation. The thymidine analog EdU was added for the last 24 h of the 7 days of cultivation. The labeling index (proportion of cells with and without proliferation signal) was higher for SgA than for SgB, with and without tilPE addition (Figures 3B–D). Twenty-two percent of counted SgA were positive for EdU incorporation in the control cultures without tilPE supplementation. A similar labeling index (around 25%) has been reported for undifferentiated SgA of zebrafish after 48 h of testis culture under basal conditions and BrdU addition for the last 6 h of culture (48). For tilPE supplemented cultures the labeling index for SgA in our study rose significantly to 34% (Figure 3B). This activation of SgA proliferation by gonadotropin and growth hormone (GH) containing tilPE is in accordance with the literature (63). A direct comparison with *ex vivo* cultures from other fish species is inherently difficult and limited, but studies on zebrafish testis cultures showed results of similar incorporation rates as in our experiments. When the endogenous androgen synthesis in zebrafish testis cultures was blocked with 25 μg/ml of the steroidogenesis inhibitor trilostane, 20% of zebrafish SgA were BrdU positive after 48 h of culture and addition of BrdU for the last 6 h of culture. This proportion rose to over 50% BrdU positive undifferentiated SgA when 100 ng/ml FSH were added. In both studies differentiated SgA behaved similarly (23, 48). In our study we have not made this distinction and recorded both groups together as SgA in our evaluation. Using a 24 h EdU incubation for the last day of culture in the present study, we intended to detect most of the proliferating cells, if one assumes that oogonia behave similarly to spermatogonia in their proliferation dynamics. Nakamura et al. (33) managed to label 60% of the fast cycling medaka oogonia after a 24 h BrdU pulse *in vivo*, another study stated the existence of slow cycling or quiescent oogonia which could not be labeled with BrdU (64). Both fast and slow cycling oogonia were speculated to have stem cell properties, and this situation could be similar to the observed slow and rapid spermatogonial renewal in fish testis (2, 65).

In our study, the labeling index for SgB was also significantly increased from 9% in the control to 25% with tilPE treatment (Figure 3B). However, we would have expected the proliferation rate for SgB to be higher than that of SgA (4, 17). This deviation from current models cannot be easily solved at this moment. Further *in vivo* experiments would be needed to determine the cell cycle times of SgA and SgB in Nile tilapia and to investigate if SgB in fact undergo the cell cycle more slowly than SgA. Another explanation for the relatively low percentage of proliferating SgB could be the method of counting these cells applied in this work. Since the Vasa signal disappears toward meiosis, there could be a bias for counting mostly early SgB. The number of cells in a cyst increases exponentially with every mitotic division. Late SgB would therefore have a much higher effect on the proliferation index than early SgB. Nevertheless, our findings showed a strong activation of SgB proliferation by tilPE. In conclusion, our analysis of spermatogonial proliferation by EdU incorporation clearly showed the activating effect of tilPE in Nile tilapia testis cultures and proved the suitability of the *ex vivo* culture system.

### hCG and tilPE Stimulate Genes Involved in Steroidogenesis but Show No Effect on Germ Cell Markers and *amh*

RT-qPCR was used to analyze the cellular response to culture with tilPE, hCG, a combination of both and the control without additives. Marker genes for Leydig cells, Sertoli cells, and the germline cells were chosen to identify those cells after 7 days of *ex vivo* culture. This approach allowed evaluating the effect of hormonal treatments on the transcriptional level and detection of possible changes in activity of certain cell types.

#### Gene Expression of the Leydig Cell Markers *stAR2* and *cyp11b2* Are Upregulated After 7 Days

**Figure 4:**
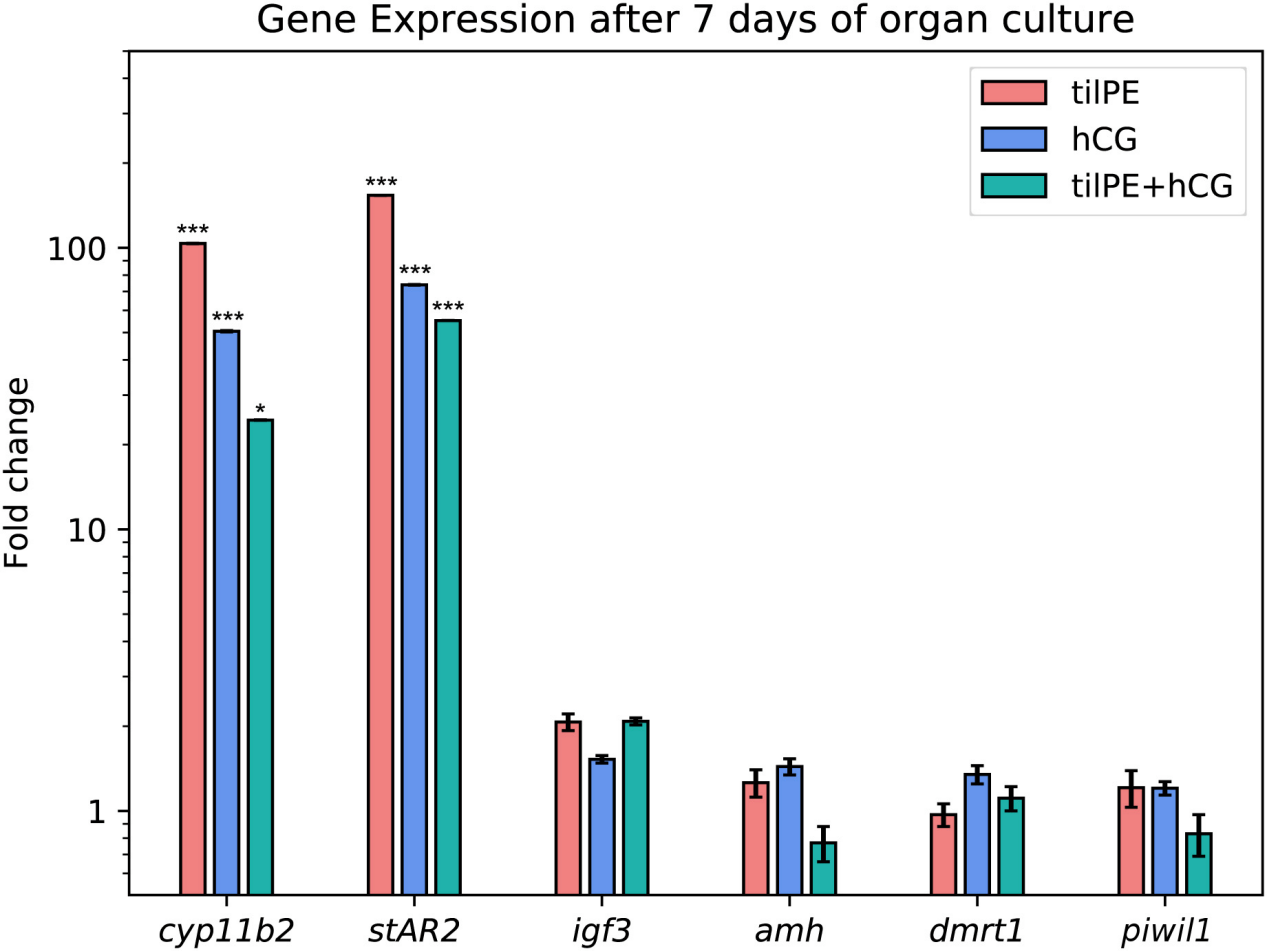
Gene expression after 7 days of organ culture. The gene expression is given as fold change compared to the untreated control. Data are represented as average fold change and SEM. tilPE treatment data includes 4 independent experiments, hCG and tilPE + hCG data include 3 independent experiments. Gene expression was measured with qPCR in three technical replicates per pooled sample. A two tailed Wilcoxon rank-sum test was used to test for significant differences between treatment and control. Significance levels were interpreted as follows: *p < 0.05, and ***p < 0.001.

A major task of the interstitial Leydig cells is the production of sex steroid hormones, namely 11-KT in male fish. For 11-KT and other steroid hormones to be synthesized, their common precursor substance, cholesterol, has to be transported from the cytoplasm to the inner mitochondrial membrane. Since the hydrophobic cholesterol cannot easily pass the aqueous inter membrane space, other molecules have to facilitate this passage. Despite years of research effort, the exact mechanisms still remain clouded, but it is agreed upon that steroidogenic acute regulatory protein (stAR) plays an important role in this rate limiting step of steroid synthesis (66). All three treatments highly stimulated the expression of *stAR* (Figure 4). In the tilPE treatment, *stAR2* expression was increased over 100-fold compared to the control. hCG alone and the combined treatment also caused an increase of about 80-fold. Two copies of the *stAR* gene have been identified in tilapia, where expression of *stAR1* was found in kidney, head kidney, testes, and in the ovary and expression of the paralogue, *stAR2*, was restricted to the gonads and was expressed more strongly in testes than in ovaries (67). When 6 month old tilapia were injected with hCG the transcription of both genes increased within a few hours, peaked at 12 h after treatment, and persisted after 24 h (67). These results are in concordance with the data produced in the present study and show that gonadotropic stimulation also occurs *ex vivo* for 7 days. Our histology results also support the observed increase in *stAR2* expression by showing well-preserved regions filled with Leydig cells (Figure 2) when tilPE was present in the culture.

The upregulation of *stAR2* by tilPE observed in our experiment concurs with a recent study in another fish species, the Spotted scat. There, recombinant gonadotropins were injected once into males and females and a rise in *stAR2* expression could be measured after 14 days (68). We assume that the induced rise of *stAR2* gene expression in Nile tilapia testis culture by tilPE, hCG and the mixture of both is due to direct effects of the gonadotropic hormones, because we could detect transcripts of both *fshr* and *lhcgr* in freshly dissected tilapia testis and also after 7 days of culture (data not shown).

The second gene which was strongly upregulated by all gonadotropin treatments in this experiment was *cyp11b2, one* of the isoforms coding for P450-11-β hydroxylase, an enzyme that plays a key role in the synthesis of 11-KT (69). The increase in gene expression was highest for the tilPE treatment (over 100-fold increase), but also significantly increased (50-fold) in the presence of hCG, a potent activator of the tilapia LH receptor (14), or the combined treatment (20-fold). These results, together with our histological observation of intact Leydig cells groups, clearly show the expected, stimulatory effect of gonadotropins on Leydig cell survival and activity over the course of the *ex vivo* cultivation. The increased expression of *cyp11b2* and *stAR2* hints at a possible endogenous synthesis of steroid hormones in the cultured tissue. As mentioned in the methods section, the dosage of LH and FSH in our organ culture experiments results in a final concentration of 430 ng/ml FSH and 780 ng/ml LH. 2–12 ng/ml LH and 2–6 ng/ml FSH have been reported in female tilapia plasma (35). For dominant tilapia males, we have measured maximum values of 37 ng/ml FSH and 20 ng/ml LH (data not shown). These amounts are similar to those reported for male sea bass (70), male Greater amberjack (71), and male Senegalese sole (72) at their peak levels. Therefore, we can consider the amounts of FSH and LH used in this study as high and sufficient to cause an activation of steroidogenesis related genes and spermatogenesis in general. We deemed such high concentrations necessary because hormones and other substances enter the tissue via diffusion in cultured conditions. The potential of both FSH and LH to induce steroidogenesis in fish is well-known (2). The effect of both hormones on steroidogenesis may be similar but there is evidence that their roles in fish spermatogenesis are not completely redundant. When monitoring the hormone profile of seasonal spawners, like trout or carp, it becomes apparent that the two substances act at different time points of gametogenesis both in males and in females. In these two species and other seasonal fish, FSH seems to be more involved in early spermatogenesis comprising the spermatogonial stages and LH plays the main role during the maturing process of future sperm (10, 73, 74). As discussed in section “7-Day Culture of Testis Explants Requires Addition of tilPE for Maintenance of Intact Testis structure” above, an influence of FSH on spermatogonial proliferation is also reported from non-seasonal fish like medaka and zebrafish (5, 26, 48, 49). Future studies should investigate differential transcriptional activation of steroidogenesis-related genes by LH and FSH.

#### Genes Associated With Spermatogonia Proliferation Are Unaffected by Culture Conditions

Fish generally have two copies of the Argonaut family gene *piwi*, named *piwil1* and *piwil2*. They are indispensable for germline integrity of both males and females during early development and adult gonads (75, 76). In Nile tilapia both *piwil* genes are more strongly expressed in the testes than in the ovary in the adult stage (77). Piwil was localized in SgA in adult zebrafish (78, 79). In another zebrafish study, *piwil* expression was used to detect the intratubular testis germ cell fraction (6). In tilapia it has been identified in both spermatogonia and spermatocytes, but not in spermatids (80). Thus, Piwil1 can be considered a germline marker in Nile tilapia testis. In tilapia testis primary cell culture and *in vivo*, hCG was found to decrease *piwil1* expression after 4 h of exposure (80), an effect also observed in female carp *in vivo* (81). In the present study, however, we could not measure an influence of hCG, tilPE, or both on *piwil1* expression compared to the control culture (Figure 4). Despite the fact that Piwil1 has been detected in different stages of germline cells, to the authors' knowledge no quantitative data about *piwil1* distribution over the different cell types has been reported for Nile tilapia to date. Since we did not observe significant differences in *piwil1* expression, a regulative influence of the gonadotropic substances tested here on tilapia *piwil1* expression could not be resolved in the present study. However, we cannot exclude the possibility that changes in cell type proportions are masking regulative effects of gonadotropins on *piwil1*.

Insulin like growth factors play a role in male and female gametogenesis in fish (82) and as mentioned above, Igf1 has been shown to enhance spermatogonia proliferation in Nile tilapia (58), for example. Igf3 is a teleost specific insulin like growth factor that is expressed in several tilapia tissues, but most prominently in the gonads (83, 84). Igf3 localization has been described in the interstitial cells of mature tilapia testes (85). The stimulatory effect of FSH on *igf3* is well-documented in zebrafish (23) and trout (73). The expression of the spermatogenesis promoting factor *igf3* increased 2-fold on average in our experiment in the tilPE and combination treatments, however, this trend was not statistically significant for either of the treatments (Figure 4).

The reason why neither hCG nor FSH containing tilPE affected *igf3* expression in the present study remains to be elucidated. Further experiments could include the use of recombinant tilapia FSH instead of tilPE to better investigate the effect of single gonadotropic substances. To unravel the regulatory relationship between FSH, *igf3*, and *amh*, it could be helpful to conduct organ culture experiments in the absence of endogenous androgens as discussed below. One study on zebrafish also briefly addressed the influence of recombinant LH on *igf3* expression (48). Two hours after injection of the hormone into male zebrafish, *igf3* expression remained unchanged. After 2-day *ex vivo* culture, LH only doubled *igf3* expression, while FSH had increased it over 20-fold. If steroidogenesis was inhibited, LH did not have an effect on *igf3*, contrary to FSH. The two gonadotropins therefore appear to act in different ways on *igf3*. This allows speculating that in the present study we might have missed the time point to detect hCG/LH regulation of *igf3*. It remains to be examined, if hCG/LH stimulation of *igf3* depends on steroids in tilapia.

#### Sertoli Cell Associated *dmrt1* and *amh* Appear Not to Be Affected by Pituitary Extract and hCG

The Sertoli cells populate the testicular tubuli together with the germ cells. They accompany single SgA or enclose cysts in which synchronous spermatogonia proliferation takes place. *dmrt1*, a conserved gene involved in determining and sustaining a male gonadal phenotype, is a marker expressed in somatic tilapia Sertoli and epithelial cells (86). In other vertebrates such as mouse (87) or zebrafish (88), *dmrt1* is also expressed in the germline. In zebrafish, *dmrt1* is assumed to play a role in self-renewal and differentiation of spermatogonia (20). Our RT-qPCR detected *dmrt1* transcript in all three samples after 7 days but there were only little, non-significant differences between the gonadotropin treatments and the control (Figure 4). Dmrt1 is probably regulated by the HPG axis via FSH. In a 4 days stationary culture experiment with testes of rainbow trout, *dmrt1* was one of the genes upregulated by FSH, but only when steroid production was not inhibited with trilostane (89). Future experiments with tilapia could therefore focus on testis exposition to steroids directly or the use of recombinant FSH instead of tilPE.

Amh is an important regulator of spermatogenesis in teleost fish and in some tilapia strains a tandem duplication (*amh*Δ*Y* and *amhY*) carries the task of a male sex determining factor (90–92). One of these copies (*amh*Δ*Y*) is not present in our fish line (Figure S7), but whether the second copy (*amhY*) exists or not in our strain is under investigation. The primers used for our qPCR would amplify all possible *amh* copies. Nevertheless, it can be assumed that our qPCR analyses detected *amh* expression mainly since *amhY* and *amh*Δ*Y* expression are restricted to early development and dropped to very low levels in adult testis (91) and can probably be neglected. In zebrafish testis Amh can be immunochemically detected in Sertoli cells surrounding SgA (19, 24). A similar location has been observed in male tilapias 180 dah (91) and in sea bass (93). First discovered to regulate spermatogonial proliferation in the Japanese eel (21), Amh acts similarly in other species, like zebrafish (19) and sea bass (93). Loss-of-function mutation of *amhrII* in male medaka (18), knock-outs of *amh* in male zebrafish (20), or either genes in female tilapia (94) lead to hyperproliferation of spermatogonia and oogonia, respectively. This suggests that *amh* expression levels should be reduced before spermatogonia are able to proliferate and differentiate. In teleosts however, the regulation of Amh appears to be species depended as reviewed by Pfennig et al. (22). One example for the diverse regulation of *amh* is the effects of androgens. 11-KT can have a stimulating, inhibitory, or no effect on *amh*, depending on the species. Expression of *amh* in zebrafish appears to be independent of androgens and estradiol-17β (23, 95) However, the available data is more consistent concerning gonadotropin controlled Amh regulation. In rainbow trout (89) and zebrafish (24) an inhibitory FSH effect on Amh is independent from the presence of androgens. In contrast, in a study using RNA-seq and RT-qPCR on zebrafish organ cultures, a significant but still weak downregulation of *amh* by FSH could only be achieved when steroid synthesis was blocked by trilostane (23).

In the present study, we did not see a decrease in *amh* mRNA abundance after 7 days of culture with hCG and tilPE, compared to the control. Only the combined treatment of hCG and tilPE showed slight, non-significant down-regulation of the *amh* transcript in some of the experiments (Figure 4 and Figure S8). We speculate that androgens can mask the effect of gonadotropins on *amh*, similar to the finding by Crespo et al. (23), as mentioned above. Another possibility for the putative unresponsiveness of *amh* could be a reaction of Sertoli cells to the culture conditions. Sertoli cells of teleost fish proliferate at high rates during the mitotic stage of spermatogenesis. In tilapia, 80% of BrdU labeled Sertoli cells have been found to be associated with spermatogonia (4). In our study, spermatogonia were stimulated by tilPE to enter mitosis. Therefore, one would expect that Sertoli cells associated with SgA and SgB also showed elevated proliferation. Thus, even if *amh* expression was downregulated, more *amh* expressing Sertoli cells would have been present, potentially masking an inhibitory effect on the gene expression level. If gonadotropin induced steroidogenesis was the main cause of spermatogonia and Sertoli cell proliferation in this study, future experiments could address the matter by measuring 11-KT release and inhibiting steroid synthesis in the culture. Such an approach could help to investigate direct effects of gonadotropins on *amh* as well.

In this paper we presented a culture system that allows studying spermatogenesis *ex vivo* over a course of 7 days. This is important because data from zebrafish experiments suggest that *amh* down-regulation by FSH takes some time. García-Lopez et al. (6) found that *amh* expression was not changed after 2 h or 2 days of testis *ex vivo* exposition to recombinant FSH or LH. FSH injection in zebrafish for example caused reduced Amh protein levels after 48 h, downregulation of *amh* mRNA *ex vivo* was measured after 3 days with 75 ng/ml FSH (24). The downregulation was even stronger after 5 days of culture. These results point out the importance of different time regimes and culture systems to fully unravel the regulation of *amh* in fish.

## Conclusion

Our data shows that 7-day organ culture is a feasible approach to study spermatogenesis in Nile tilapia. Histology of immuno-stained samples revealed that the testis slices only maintained their structural integrity completely when tilPE was added to the medium. Tilapia pituitary extract also promoted the proliferation of both SgA and SgB. Thus, we conclude that in *ex vivo* cultures, components of tilPE, such as FSH, LH, and GH, are crucial for inducing SgA proliferation and facilitating the transition of SgA to SgB. qPCR analyses suggest that both tilPE and hCG stimulate the expression of steroidogenesis related genes like *stAR2* and *cyp11b2*, which is in concordance with our observation that tilPE supplementation preserves testis structures and spermatogenesis in culture. However, tilPE and hCG have no effect on transcript levels of other spermatogenesis related genes like *igf3* and *piwil1* and on Sertoli cell markers *amh* and *dmrt1*. Our organ culture system in combination with tilPE and hCG provides a powerful tool for investigating the endocrine control of spermatogenesis in the testis. Furthermore, the use of an organ culture system, allows the study of more complex phenomena than primary cell culture while also avoiding direct hormonal manipulation of live animals. Future experiments will address the application of steroids, steroidogenesis inhibitors, or any other substance with an influential role in spermatogenesis.

## Data Availability Statement

All datasets generated for this study are included in the article/Supplementary Material.

## Author Contributions

FP, MT, AF, and BL-S contributed conception and design of the study. MT, MV, FP, and KH conducted the experiments and analyzed the data. KS performed PCR analyses for detection of *amh*Δ*Y*. MT and FP wrote the first draft of the manuscript. MT, FP, AF, KH, and BL-S were involved in manuscript preparation. All authors contributed to manuscript revision, read, and approved the submitted version.

This work was supported by the use of a microscope procured by the Applied Zoology lab under the Excellence Initiative of the DFG (Zukunftskonzept). We are grateful to all members and students of the Froschauer group for helping hands in fish maintenance.

## Conflict of Interest

The authors declare that the research was conducted in the absence of any commercial or financial relationships that could be construed as a potential conflict of interest.
