# Supplementary figures and images for: An ex vivo Approach to Study Hormonal Control of Spermatogenesis in the Teleost Oreochromis niloticus
Source: Front Endocrinol (Lausanne). 2020 Jul 10;11:443. doi: 10.3389/fendo.2020.00443 (PMC7366826; doi:10.3389/fendo.2020.00443)

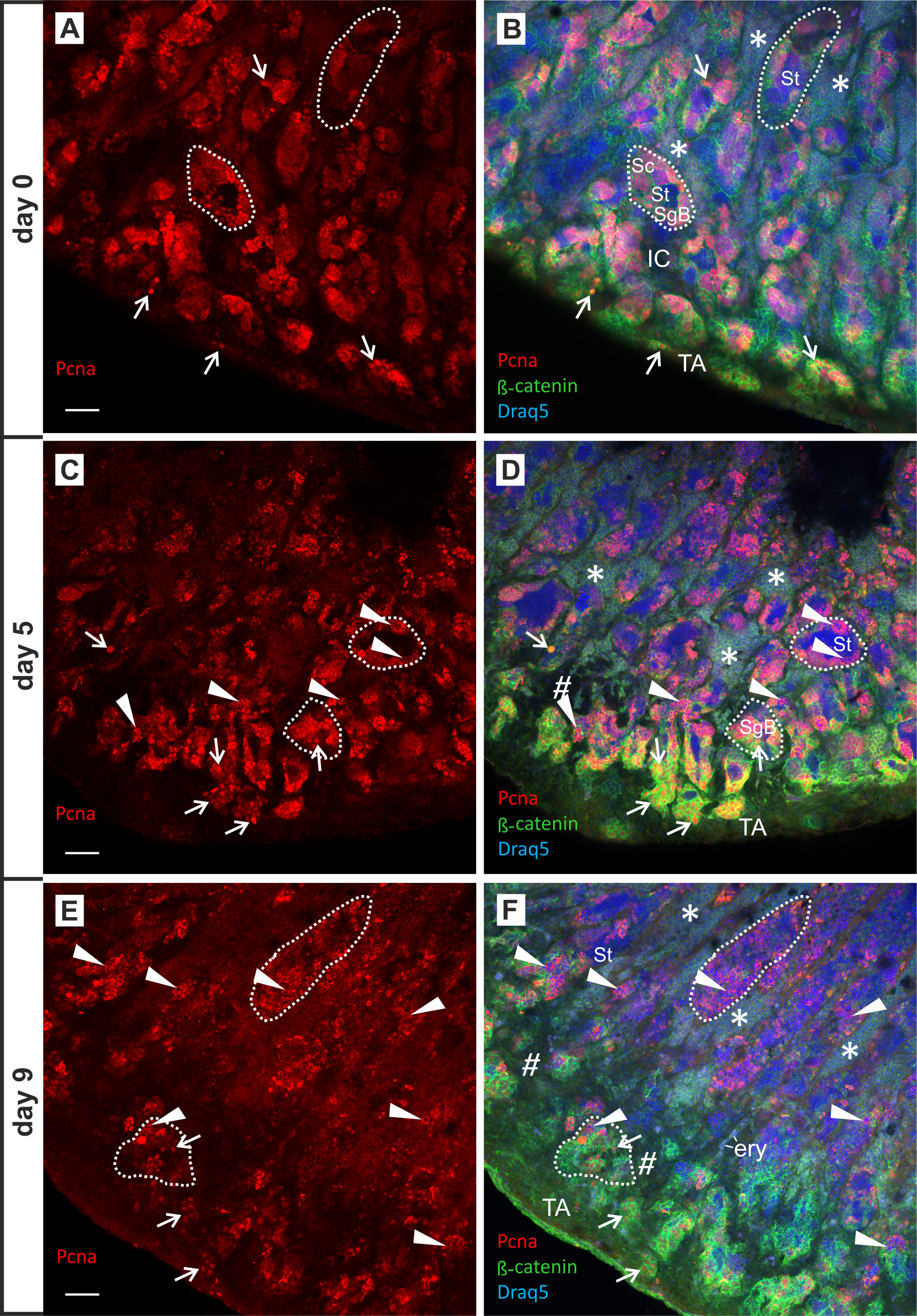

Supplement: Supplementary file 1 [file Image_1.TIF]

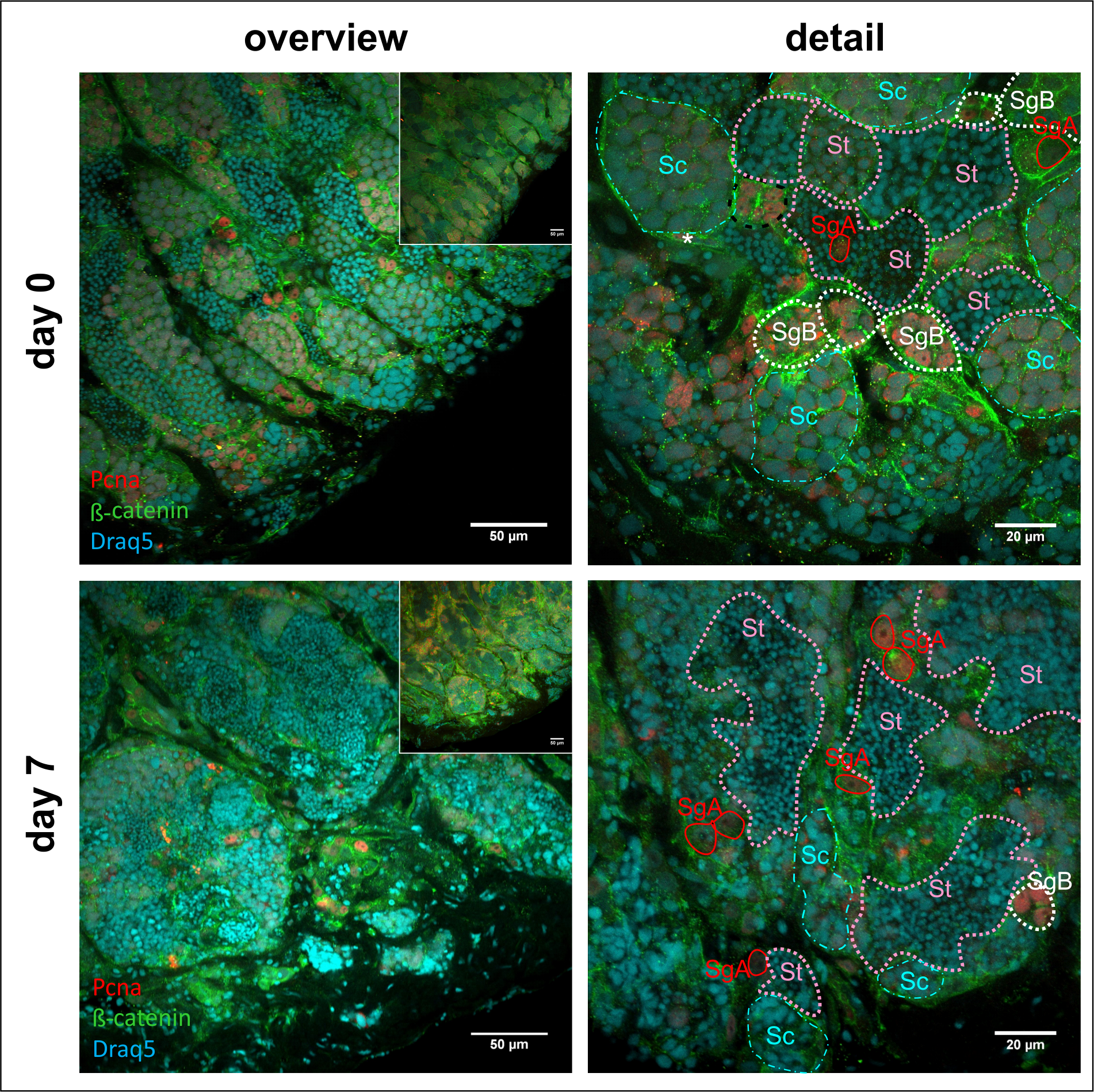

Supplement: Supplementary file 2 [file Image_2.TIF]

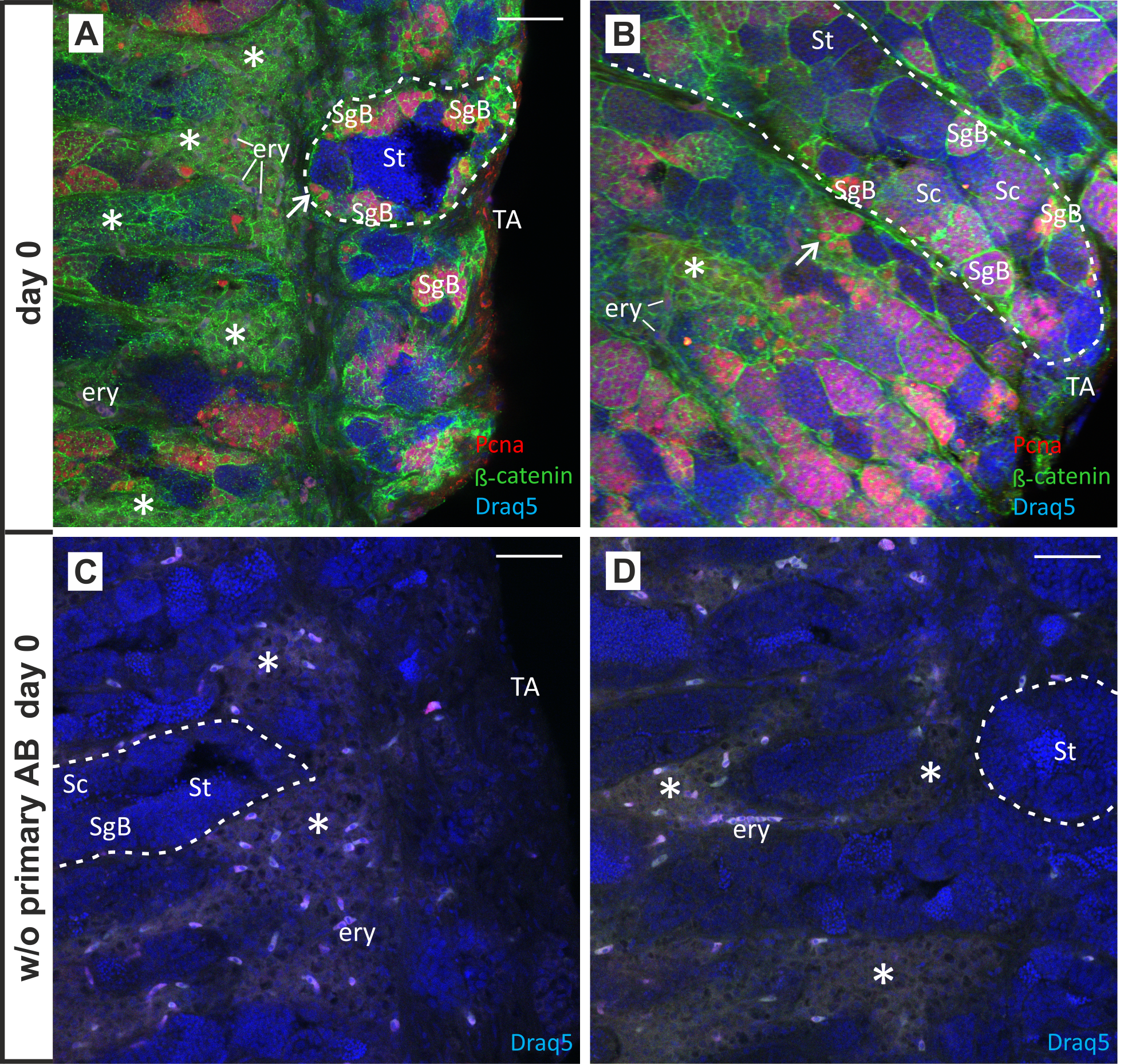

Supplement: Supplementary file 3 [file Image_3.TIF]

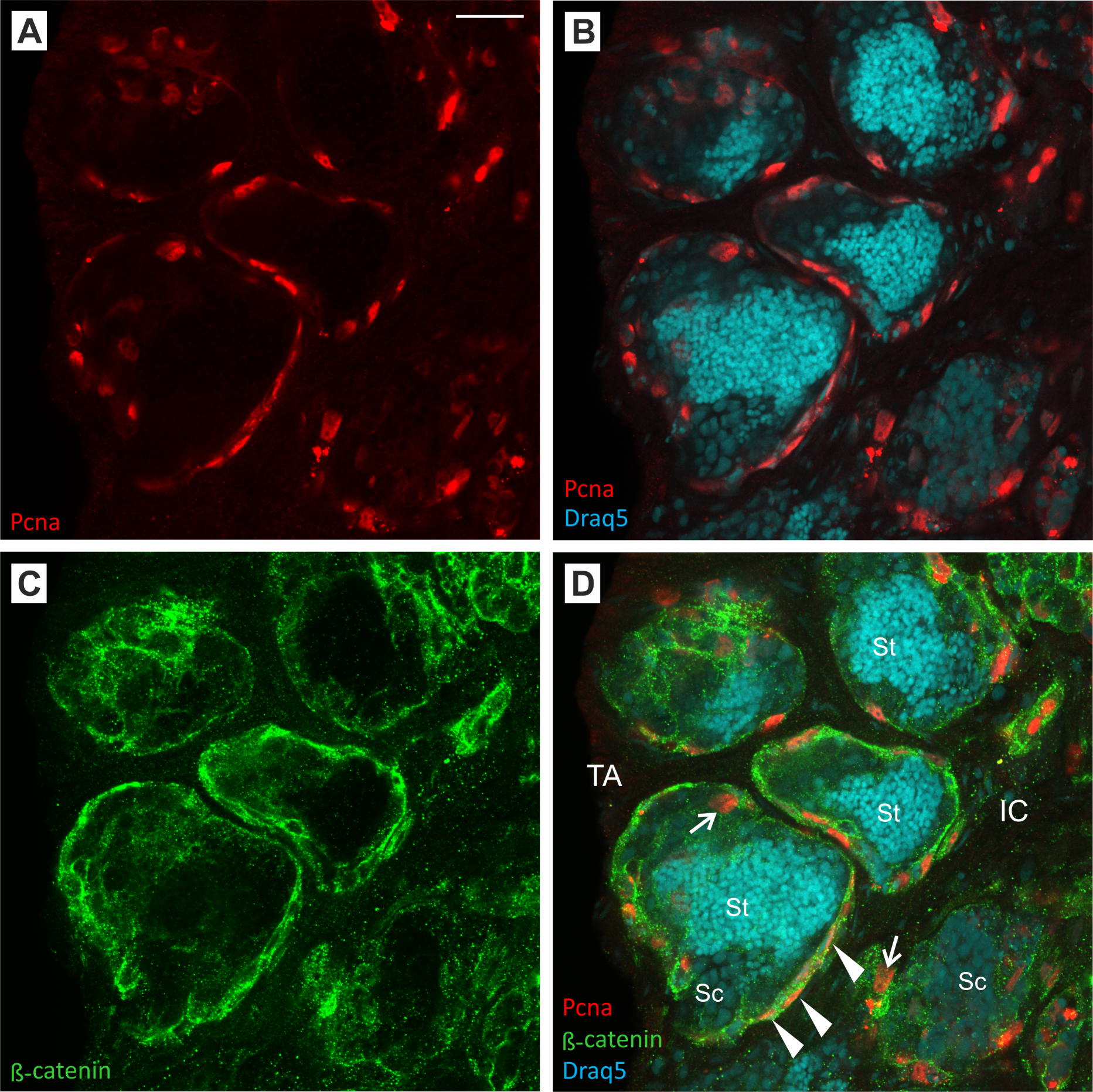

Supplement: Supplementary file 4 [file Image_4.TIF]

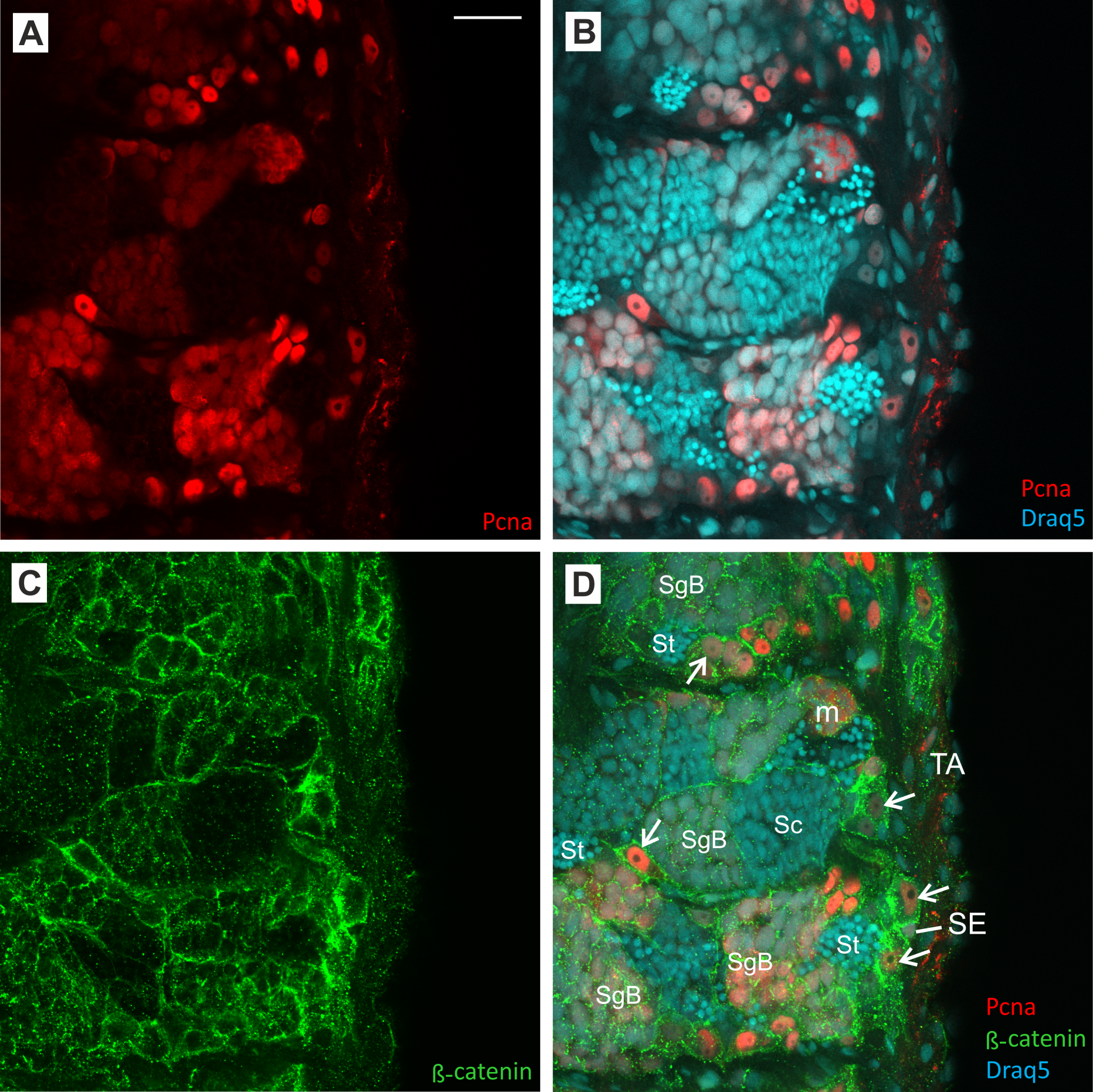

Supplement: Supplementary file 5 [file Image_5.TIF]

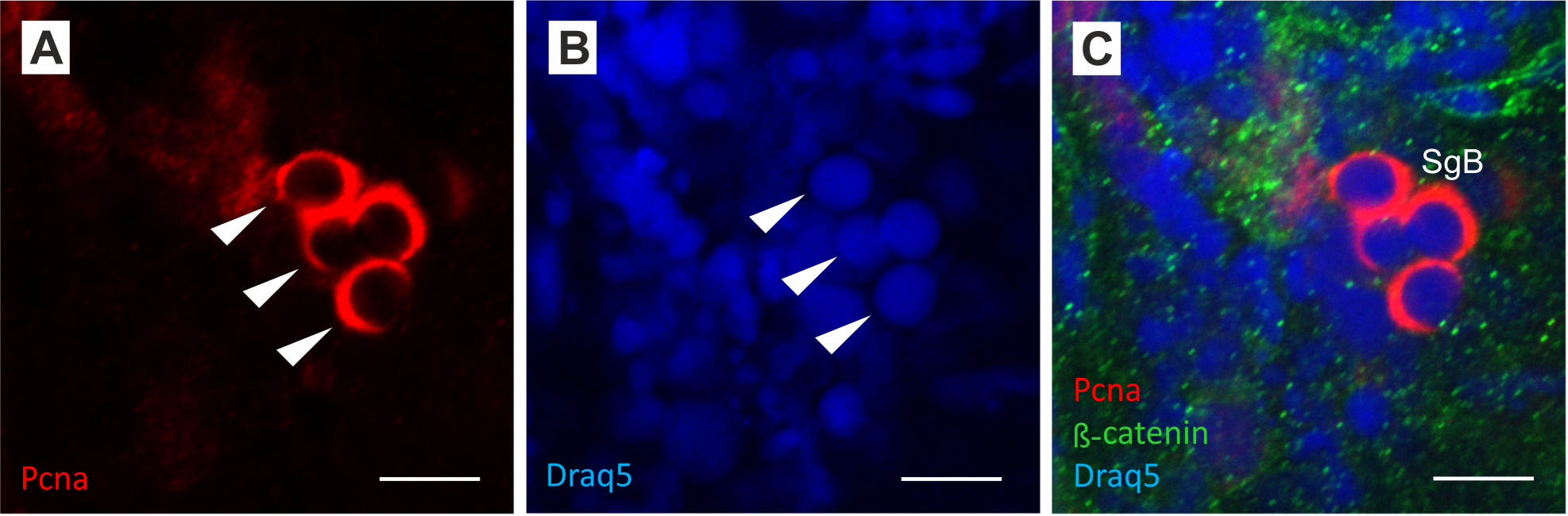

Supplement: Supplementary file 6 [file Image_6.TIF]

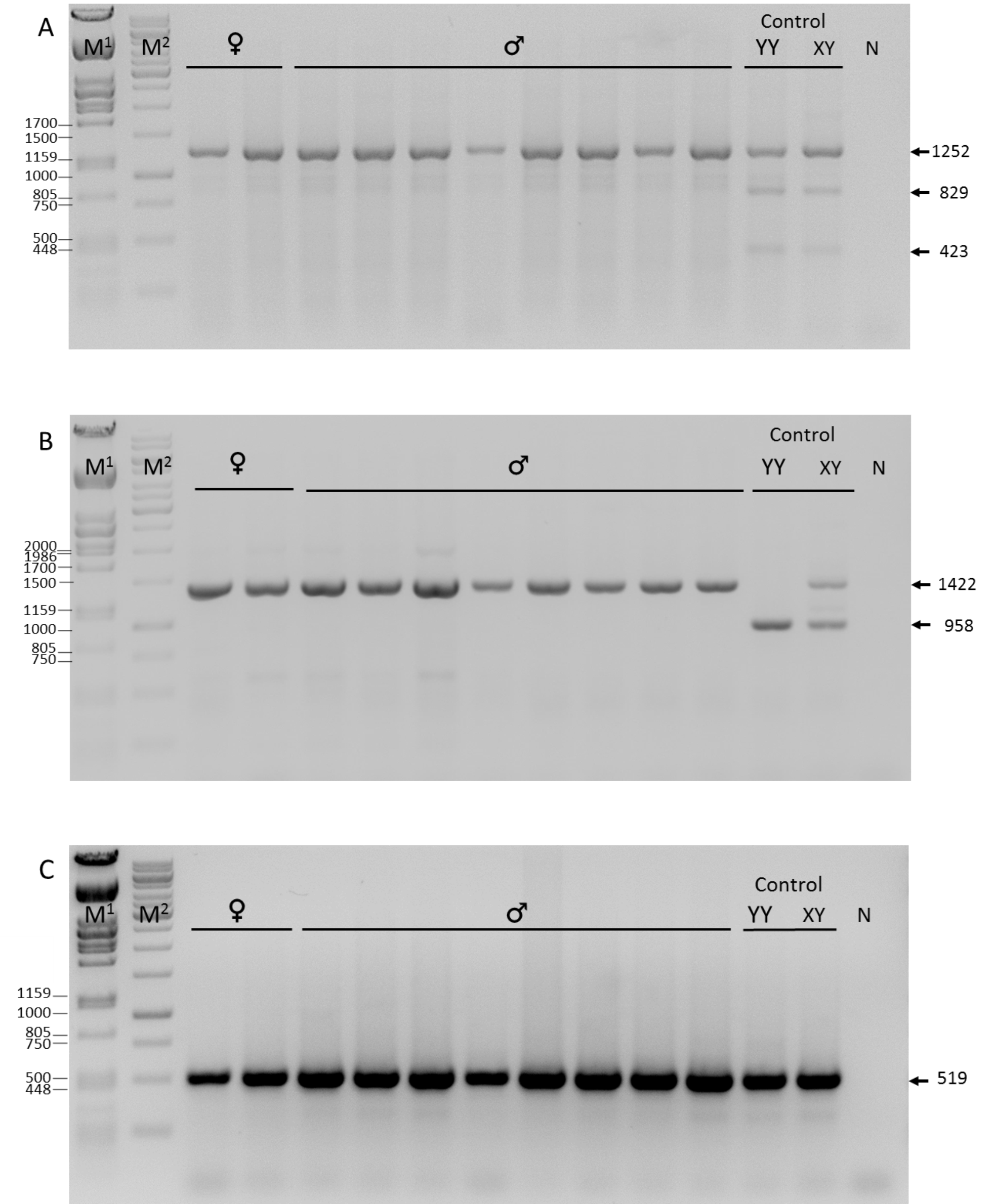

Supplement: Supplementary file 7 [file Image_7.TIF]

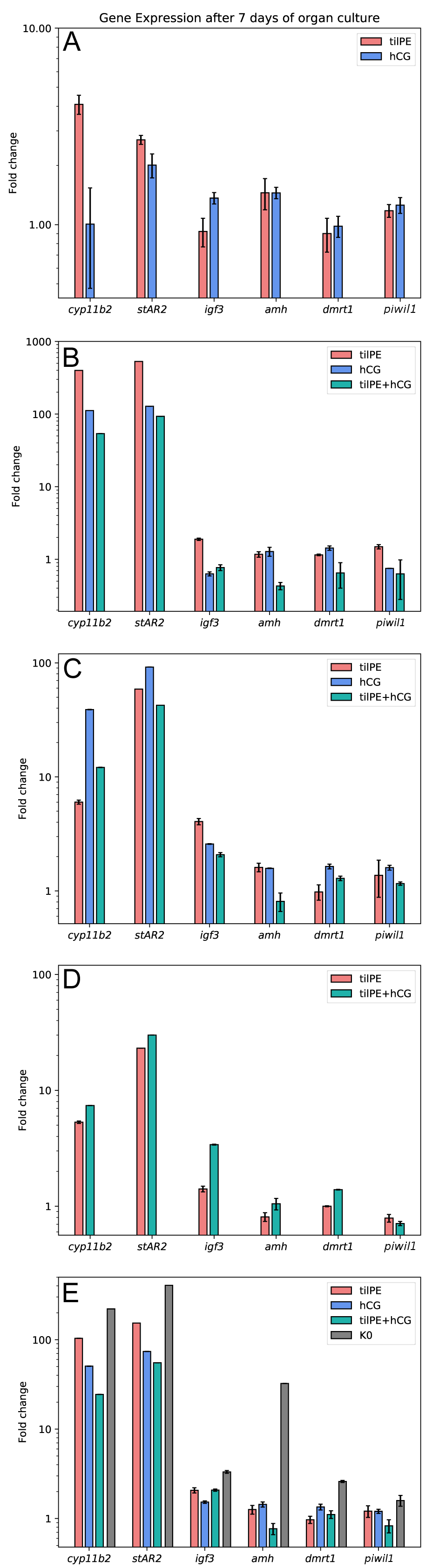

Supplement: Supplementary file 8 [file Image_8.TIF]
