# Supplementary material for: An ex vivo Approach to Study Hormonal Control of Spermatogenesis in the Teleost Oreochromis niloticus
Source: Front Endocrinol (Lausanne). 2020 Jul 10;11:443. doi: 10.3389/fendo.2020.00443 (PMC7366826; doi:10.3389/fendo.2020.00443)
Supplement: Supplementary file 9 [file Table_1.docx]

Table S1: Experimental animals used in this study.

A and B refer to biological replicates.

| **Experiment** | **Section in the manuscript** | **Fish** | **Age** | **GSI** | **social status** |
| --- | --- | --- | --- | --- | --- |
| **Gene expression and EdU incorporation** | 3.2/3.3 | 1A | 13 months | 0.32 | subordinate |
|  | 3.2/3.3 | 2A | 15 months | 0.18 | subordinate |
|  | 3.2/3.3 | 2B | 15 months | 0.14 | subordinate |
|  | 3.2/3.3 | 3A | 21 months | 0.17 | subordinate |
|  | 3.2/3.3 | 3B | 21 months | 0.24 | subordinate |
|  | 3.2/3.3 | 4A | 31 months | 0.10 | subordinate |
|  | 3.2/3.3 | 4B | 31 months | 0.12 | subordinate |
| **Static culture (S1)** | 3.1 | 5 | 55 months | 0.21 | dominant |
| **Maintenance of testis structure and function** | 3.1 | 6 | 34 months | 0.13 | subordinate |
|  | 3.1 | 7 | 34 months | 0.18 | subordinate |
