# Supplementary material for: An ex vivo Approach to Study Hormonal Control of Spermatogenesis in the Teleost Oreochromis niloticus
Source: Front Endocrinol (Lausanne). 2020 Jul 10;11:443. doi: 10.3389/fendo.2020.00443 (PMC7366826; doi:10.3389/fendo.2020.00443)
Supplement: Supplementary file 10 [file Table_2.docx]

Table S2: Primers used in this study.

| **Category** | **Primer** | **Sequence (5‘ to 3‘)** | **Product** | **Slope** | **Efficiency (%)** | **Error** | **R2** | **Source** |
| --- | --- | --- | --- | --- | --- | --- | --- | --- |
| **Housekeeping gene** | Q-18S-F | TGGCAAATACAGCCAGGTCC | 106 bp | -3.559 | 90.989 | 0.034 | 0.999 | This work |
|  | Q-18S-R | CCAGAAGTGCCTGAGACCAC |  |  |  |  |  |  |
| **Sertoli cell** | Q-dmrt-F | CGGCCCAGGTTGCTCTGAG | 136 bp | -3.408 | 96.537 | 0.069 | 0.995 | Jiang et al. ( 2016) |
|  | Q-dmrt-R | AACGTGAAGACAGCGTGAAG |  |  |  |  |  | This work |
|  | Q-amh-19/20-F | AACGTACGACGCGCAAAG | 215 bp | -3.126 | 108.866 | 0.147 | 0.974 | Pfennig et al. (2012) |
|  | Q-amh-19/20-R | TCTCGATGTGGGAGTTGAGC |  |  |  |  |  |  |
| **Leydig cell** | tcyp11b2-F | CAAAGAAGTCCTCAGGTTGTACCCA | 103 bp | -3.194 | 105.653 | 0.084 | 0.992 | Ijri et al. (2008) |
|  | tcyp11b2-R | GGACCAAAGTTCCAGCAGGTATGT |  |  |  |  |  |  |
|  | Q-Star2-F | GTTTGAGGTTGCTGAGAGTAATGGT | 169 bp | -3.159 | 107.27 | 0.047 | 0.997 | Yu et al. (2013) |
|  | Q-Star2-R | TTGCTGTATGCTTGGGTTCC |  |  |  |  |  |  |
| **Germline** | Q-Piwi1-F | TCTTTCGCATCCTGTCCCG | 131 bp | -3.328 | 99.727 | 0.041 | 0.998 | Xiao et al. (2013) |
|  | Q-Piwi1-R | CCGTGCTGAGCCAAGTGTTT |  |  |  |  |  |  |
| **Spermatogonia proliferation** | Q-Igf3-F | CAGACACTCCAGGTGCTGTGTG | 168 bp | -3.301 | 100.887 | 0.092 | 0.988 | Li et al. (2012) |
|  | Q-Igf3-R | CAAGCCTTTACGTAAATAGATTCC |  |  |  |  |  |  |
| **Control cDNA synthesis** | β-actin F | GATCCGGTATGTGCAAGG | 317 bp cDNA,  519 bp gDNA | Conventional PCR. Allows discrimination between cDNA and gDNA | | | | This work |
|  | β-actin R | CTTCTCCCTGTTGGCTTTGG |  |  |  |  |  |  |
