# Supplementary material for: An ex vivo Approach to Study Hormonal Control of Spermatogenesis in the Teleost Oreochromis niloticus
Source: Front Endocrinol (Lausanne). 2020 Jul 10;11:443. doi: 10.3389/fendo.2020.00443 (PMC7366826; doi:10.3389/fendo.2020.00443)
Supplement: Supplementary file 11 [file Table_3.docx]

Table S3: Wilcoxon rank sum test with continuity correction for qPCR evaluation.

| **Category** | **gene** | **hCG vs control** | | **tilPE vs. control** | | **hCG+tilPE vs. control** | |
| --- | --- | --- | --- | --- | --- | --- | --- |
|  |  | **w** | **p-value** | **w** | **p-value** | **w** | **p-value** |
| **Sertoli cell** | ***dmrt1*** | 35 | 0.6587 | 64 | 0.6707 | 38 | 0.8633 |
|  | ***amh*** | 40 | 1 | 65 | 0.7125 | 33 | 0.5457 |
| **Leydig cell** | ***cyp11b2*** | 73 | 0.002756 | 126 | 0.001115 | 63 | 0.5031 |
|  | ***stAR2*** | 68 | 0.0009872 | 114 | 0.002185 | 64 | 0.0001554 |
| **Germline** | ***piwi1*** | 24 | 0.6943 | 62 | 0.9487 | 34 | 0.8785 |
| **Spermatogonia proliferation** | ***igf3*** | 25 | 0.536 | 88 | 0.3864 | 47 | 0.3213 |
