# Supplementary material for: An ex vivo Approach to Study Hormonal Control of Spermatogenesis in the Teleost Oreochromis niloticus
Source: Front Endocrinol (Lausanne). 2020 Jul 10;11:443. doi: 10.3389/fendo.2020.00443 (PMC7366826; doi:10.3389/fendo.2020.00443)
Supplement: Supplementary file 12 [file Data_Sheet_1.docx]

Supplementary Material - Figure legends

**FIGURE S1: Confocal images of immunolabeled vibratome sections of testis explants in static culture after 5 and 9 days of cultivation in comparison to the normally developed testis (day 0).** Scale bares: 20 mm valid for all pictures. **(A, C, E)** Pcna: red fluorescence. **(B, C, F)** Pcna (red) and β-catenin (green), DNA stained by Draq5 (as false color blue). Overlay of red, green and blue color. Normal testis structure **(A, B)** shows compact spermatogenic tubules (exemplarily labeled by white dotted line) with distinct Leydig cells groups in the interstitium (exemplarily labeled by asterisks) between tubules. Interstitial parts without Leydig cells marked as **IC** exemplarily. **TA** – tunica albuginea. **St** – spermatids. Some **SgA** are marked by white arrows in (**A**). All stages of spermatogenesis were present at the beginning of culture, premeiotic stages (SgA, SgB and early spermatocytes (**Sc**)) occurred with and without Pcna signal. **(C, D)** After 5 days of culture an increase of the interstitial space without Leydig cells occurred (labeled by **#** exemplarily). SgA can be observed (**white arrows**) but SgB with nuclear Pcna were reduced and many cysts with cytoplasmic Pcna localization appeared instead (**white arrowheads**). Such a cytoplasmic Pcna pattern was not observed at the beginning of cultivation at day 0. **(E, F)** After 9 days of static culture (media change once during incubation) only SgA with a nuclear Pcna signal were detected. The increase of the interstitial area continued. Spermatogenic tubules are still present but the proportion of SgB seems to be massively reduced and many further differentiated germ line cells show the unusual cytoplasmic Pcna signal. Spermatids can still be seen. Erythrocytes (**ery**) appear in a light grey-blue coloration around their blue nucleus and are marked exemplarily in (**F**). They can be found at every time point (**B, D, F**). Postmeiotic germ line cells have no Pcna and appear in blue by Draq5 stained nuclei inside of the tubules. Long term static culture without tilPE stimulus revealed degenerative changes at least from day 5 of culture. SgA with strong β-catenin staining and Pcna nuclear signal remain until end of culture at day 9. Interestingly, intratubular cells (preferentially cysts with SgB) with cytoplasmic Pcna signal were highly increased during the course of culture. This is the cell type which was lost in our flow-through cultures lacking tilPE stimulus.

**FIGURE S2: Confocal images of Pcna (red), β-catenin (green) and DNA (cyan) labeled vibratome sections of Nile tilapia testes before culture (day 0) and after 7 days culture without gonadotropic supplementation (day 7).** Pictures on the left show an overview in two different magnifications (lowest magnification in the small inserts); scale bars: 50 µm. Right side shows a higher magnification and typical germ line cells and germ cell cysts were labeled by a color code: **SgA** – red line and letters, **SgB** – white letters and white dashed lines, **Sc** (Spermatocytes) – light-blue letters and light-blue dot-dashed line, **St** (spermatids) – pink letters and pink dashed line; scale bars: 20 µm. After 7 days culture the interstitium appears enlarged but without the typical presence of Leydig cells as it is the case in an active testis. Further, Pcna positive cysts with SgB are reduced notably, whereas later stages and SgA seems to be intact. At both time points, SgA display different intensities of Pcna labeling.

**FIGURE S3: Confocal images of Pcna (red) and β-catenin (green) immune-labeled vibratome sections of Nile tilapia testis and associated controls where the primary antibodies were omitted. (A), (B)** Testis sections from two different explants (day 0). Large groups of Leydig cells are indicated by an asterisk (*****). Erythrocytes inside of these groups appear in a light grey-blue coloration. Spermatogenic tubules have cysts with cells of all stages of spermatogenesis. Wide Pcna labeling of single **SgA** (exemplary shown by a white arrow) and cysts consisting of **SgB** and Spermatocytes (**Sc**) occurred. Postmeiotic germ line cells show no Pcna and appear in blue by Draq5 stained nuclei inside of the tubules. One spermatogenic tubule is labeled by a white dotted line exemplary. β-catenin labeling colors large areas of the testis and highest signal intensity were observed for spermatogonia and Leydig cells. **(C), (D)** Testis sections from two different explants (day 0) show no background signal at the controls without primary antibodies. Cell nuclei are stained by DNA Dye Draq5 – shown in false color in blue. Leydig cells groups (*) appear in faint grey-brownish color caused by light background fluorescence in the red and green channel. Nucleated erythrocytes (**ery**) can be easily detected by their unspecific fluorescence in all channels, resulting in a typical grey-blue appearing cytoplasm around the blue stained nucleus. **TA** – tunica albuginea. **St** – spermatids (with the smallest nuclear diameter around 2 to 3,4 µm inside the tubules). Scale bars 50 µm.

**FIGURE S4: Confocal images of a Pcna (red), β-catenin (green) and DNA (cyan) labeled vibratome section of partly degenerated Nile tilapia testis after 7 days culture without gonadotropic supplementation. (A)** red channel (Pcna only). Scale bar: 20 µm. **(B)** red and cyan channel (Pcna and DNA). **(C)** green channel (β-catenin only). **(D)** Merge of B and C (red, green and cyan channels). Marks shown in (D) are valid for all pictures. The tunica albuginea (**TA**) is orientated to the left side. Spermatids (**St**) and spermatocytes (**Sc**) are the most abundant germ line cell type whereas SgB are not seen in that sample and only some SgA can be observed (**white arrows**). **White arrowheads** illustrate Pcna positive cell nuclei of putative myoid cells which normally are not labeled to such extend by Pcna in the tubule wall. Furthermore, an enlarged interstitial space (**IC**), here not mainly filled with Leydig cell as it is the case in a normal developed testis, can be observed. Since strong β-catenin labeling is normally associated with SgA and SgB, the β-catenin stained parts inside of a tubule seem reduced and tubule walls display enhanced β-catenin and Pcna signal intensity. Changed testis morphology and tubular organization becomes obvious in comparison to images from normally developed testis (Figure S5 in the Supplementary Material).

**FIGURE S5: Confocal images of a Pcna (red), β-catenin (green) and DNA (cyan) labeled vibratome section of typical Nile tilapia testis after 7 days culture with tilPE supplementation. (A)** red channel (Pcna only). Scale bar 20 µm. **(B)** red and cyan channel (Pcna and DNA). **(C)** green channel (β-catenin only). **(D)** Merge of B and C (red, green and cyan channels). Marks shown in (**D**) are valid for all pictures. The tunica albuginea (**TA**) is orientated to the right side and is interconnected with the interstitial tissue (groups of Leydig cells are not included in the area shown). Many single Pcna labelled germ line cells (**SgA** **-** white arrows) and cysts with **SgB** are shown. Cysts containing spermatocytes (**Sc**) and spermatids (**St**) are marked exemplarily. One example of a typical Sertoli cell (**SE**) is also highlighted. Cyst with mitotic SgB (**m**).

**FIGURE S6: Example of an unusual cytoplasmic Pcna localization in SgB** (exemplarily labeled by flat triangles). Confocal images of Pcna (red), β-catenin (green) and DNA (blue) labeled vibratome sections of Nile tilapia testes after 7 days culture without tilPE supplementation (= control at day 7). (**A**) red channel only (Pcna). (**B**) DNA stain only (by Draq5 in false color as blue). (**C**) Overlay of all three signals. Scale bars: 10 µm.

**FIGURE S7: Agarose gels showing PCR reactions for detection of *amh∆Y* in the used Nile tilapia strain.** **(A)** PCR products after subsequent *Taq^α^I* digest. With primers SW_amh_13F20_F1-5’- GTAAAACGACGGCCAGTGCTGTGTGCATTTCAGGAGA and SW_amh_13R27_R1-5’-GGAAACAGCTATGACCATGCAGCCAAGCTCACACACACT a 1252 bp product was amplified from *amh* gene(s). Yellow labelled parts of the primer sequence are specific M13F or M13R sequences and not part of the *amh* gene. The *Taq^α^I* enzyme (T/CGA) cuts close to (ATGTC) of the *amh∆Y*-specific 5bp insertion in exon 6 and subsequent *Taq^α^I* digest indicate the presence of the *amh∆Y* copy by the appearance of additional bands with 829 bp and 423 bp in size. These bands appear using XY genomic DNA from the Manzala population as a control and disappear using genomic DNA from an YY individual (Müller-Belecke and Hörstgen-Schwark, 2007; genomic YY and XY DNA’s kindly provided by Stephan Wessels). 2 females (**♀**) and 8 individual male samples (♂) are shown in comparison to the control strain and no digestion of the PCR product occurred in the samples from our Nil tilapia breeding stock. **(B)** PCR amplification with primers specific for the *amh* and *amh∆Y* promoter region according to Li *et al*., 2015. With primers Til-SP-F1-5’-ATGGCTCCGAGACCTTGACTG and Til-SP-R1-5’-CAGAAATGTAGACGCCCAGGTAT a 1422 bp amplicon indicates the *amh(a)* promoter region and a PCR-product of 958 bp indicates the *amh∆Y* specific region. These primers cannot prime with *amhY* promoter region. All samples with an *amh(a)* copy show the 1422 bp amplicon. The 958 bp band appears for the samples with the Manzala Y chromosome only in the controls. For the YY control the 1422 bp band is absent. **(C)** Control PCR with the *β-actin* gene showing template integrity. Primers betaAktin F1-5’-GATCCGGTATGTGCAAGG and betaAktin R1-5’-CTTCTCCCTGTTGGCTTTGG were used and generate a 519 bp amplicon in all samples. DNA size standards shown in all pictures: M^1^ - λ-PstI; M^2^ - 1 kb DNA ladder. PCR conditions for (A) and (C): standard PCR according to the manufactures instructions for GoTaq® Polymerase (Promega). 50 ng template, 0,1 units GoTaq® Polymerase, 1,7 mM MgCl_2_ f.c.; annealing temperature 54°C and 36 cycles for (A) and annealing temperature 60°C and 32 cycles for (C). PCR conditions for (B): standard PCR according to the manufactures instructions for Phusion® High-Fidelity DNA Polymerase (NEB). 10 ng template, 0,4 units Phusion® High-Fidelity DNA Polymerase, 1,5 mM MgCl_2_ f.c.; annealing temperature 62°C and 31 cycles.

**References**

Müller-Belecke, A. and Hörstgen-Schwark, G. (2007). A YY-male *Oreochromis niloticus* strain developed from an exceptional mitotic gynogenetic male and growth performance testing of genetically all-male progenies. *Aquaculture Research* 38, 773-775. doi:10.1111/j.1365-2109.2007.01712.x

Li, M.H., Sun, Y.L., Zhao, J.E., Shi, H.J., Zeng, S., Ye, K., et al. (2015). A Tandem Duplicate of Anti Mullerian Hormone with a Missense SNP on the Y Chromosome Is Essential for Male Sex Determination in Nile Tilapia, Oreochromis niloticus. *Plos Genetics* 11(11). doi: ARTN e1005678 10.1371/journal.pgen.1005678.

**FIGURE S8: Parallel experiments used for gene expression evaluation after 7 days of organ culture.** (**A-D**) The gene expression is given as fold change compared to the untreated control. Data are represented as average fold change and SEM. Every picture represents the results of one of the four replicates combined in Figure 4. (**E**) Combined gene expression in comparison to K0 (K0 = cDNA prepared from testis tissue before culture directly after dissection at the beginning of the experiment; gene expression at K0 was measured for experiments B, C and D and the average values are given).
